# Synchronous Infra-Slow Oscillations Organize Ensembles of Accessory Olfactory Bulb Projection Neurons into Distinct Microcircuits

**DOI:** 10.1523/JNEUROSCI.2925-19.2020

**Published:** 2020-05-20

**Authors:** Chryssanthi Tsitoura, Sebastian T. Malinowski, Julia Mohrhardt, Rudolf Degen, Brett T. DiBenedictis, Yuan Gao, Katja Watznauer, Kira Gerhold, Maximilian Nagel, Monika Weber, Markus Rothermel, Ileana L. Hanganu-Opatz, Yoram Ben-Shaul, Ian G. Davison, Marc Spehr

**Affiliations:** ^1^Department of Chemosensation, Institute for Biology II, RWTH Aachen University, Aachen, D-52074, Germany; ^2^Research Training Group 2416 MultiSenses-MultiScales, RWTH Aachen University, Aachen, D-52074, Germany; ^3^International Research Training Group 2150 The Neuroscience of Modulating Aggression and Impulsivity in Psychopathology, RWTH Aachen University, Aachen, D-52074, Germany; ^4^Department of Biology, Boston University, Boston, Massachusetts 02115; ^5^Neuromodulation Group, Institute for Biology II, RWTH Aachen University, Aachen, D-52074, Germany; ^6^Developmental Neurophysiology, Center for Molecular Neurobiology, University Medical Center Hamburg-Eppendorf, Hamburg, D-20251, Germany; ^7^Faculty of Medicine, Department of Medical Neurobiology, The Hebrew University of Jerusalem, Jerusalem 91120, Israel

**Keywords:** accessory olfactory bulb, chemosensory coding, ensemble formation, mitral cells, neural oscillations, vomeronasal system

## Abstract

The accessory olfactory system controls social and sexual behavior. In the mouse accessory olfactory bulb, the first central stage of information processing along the accessory olfactory pathway, projection neurons (mitral cells) display infra-slow oscillatory discharge with remarkable periodicity. The physiological mechanisms that underlie this default output state, however, remain controversial. Moreover, whether such rhythmic infra-slow activity patterns exist in awake behaving mice and whether such activity reflects the functional organization of the accessory olfactory bulb circuitry remain unclear. Here, we hypothesize that mitral cell ensembles form synchronized microcircuits that subdivide the accessory olfactory bulb into segregated functional clusters. We use a miniature microscope to image the Ca^2+^ dynamics within the apical dendritic compartments of large mitral cell ensembles *in vivo*. We show that infra-slow periodic patterns of concerted neural activity, indeed, reflect the idle state of accessory olfactory bulb output in awake male and female mice. Ca^2+^ activity profiles are distinct and glomerulus-specific. Confocal time-lapse imaging in acute slices reveals that groups of mitral cells assemble into microcircuits that exhibit correlated Ca^2+^ signals. Moreover, electrophysiological profiling of synaptic connectivity indicates functional coupling between mitral cells. Our results suggest that both intrinsically rhythmogenic neurons and neurons entrained by fast synaptic drive are key elements in organizing the accessory olfactory bulb into functional microcircuits, each characterized by a distinct default pattern of infra-slow rhythmicity.

**SIGNIFICANCE STATEMENT** Information processing in the accessory olfactory bulb (AOB) plays a central role in conspecific chemosensory communication. Surprisingly, many basic physiological principles that underlie neuronal signaling in the AOB remain elusive. Here, we show that AOB projection neurons (mitral cells) form parallel synchronized ensembles both *in vitro* and *in vivo*. Infra-slow synchronous oscillatory activity within AOB microcircuits thus adds a new dimension to chemosensory coding along the accessory olfactory pathway.

## Introduction

In rodents, the accessory olfactory system controls conspecific chemical communication during social interactions (Dulac and Torello, 2003; Brennan and Zufall, 2006; Tirindelli et al., 2009; Mohrhardt et al., 2018). Sensory neurons in the vomeronasal organ detect behaviorally relevant chemosignals and relay this information to the accessory olfactory bulb (AOB). As its sole projection neurons, AOB mitral/tufted cells (AMCs) transfer information to amygdalar and hypothalamic nuclei (Stowers and Logan, 2010). Despite recent insights into important organizational aspects of connectivity, sensory input, and integration in the AOB (Del Punta et al., 2002; Ma and Lowe, 2004; Sugai et al., 2005; Wagner et al., 2006; Castro et al., 2007; Ben-Shaul et al., 2010; Smith and Araneda, 2010; Hovis et al., 2012; Leszkowicz et al., 2012; Shpak et al., 2012; Tolokh et al., 2013; Hammen et al., 2014), a detailed conceptual understanding of the physiological principles that govern AMC sensory processing is lacking.

Unlike main olfactory bulb mitral cells, AMCs receive input from multiple glomeruli (Takami and Graziadei, 1991; Larriva-Sahd, 2008; Yonekura and Yokoi, 2008) and spontaneous AMC activity does not follow the breathing rhythm. By contrast, rather sparse and Poisson-like discharge has been recorded electrophysiologically from both anesthetized (Hendrickson et al., 2008; Ben-Shaul et al., 2010) and awake behaving mice (Luo et al., 2003). More recently, however, several groups have shown that a subpopulation of AMCs displays slow and periodic bursts of “idle” state activity (Gorin et al., 2016; Vargas-Barroso et al., 2016; Zylbertal et al., 2017). This infra-slow oscillatory resting state may be coordinated by a group of intrinsically rhythmogenic AMCs (Gorin et al., 2016) and/or it might be generated by network interactions (Zylbertal et al., 2017). Unlike stereotyped oscillations in the main olfactory system, which fall into relatively discrete frequency bands (Kay, 2015), infra-slow AMC oscillations appear more heterogeneous, raising the possibility that they functionally bind particular neuronal ensembles (Gorin et al., 2016; Zylbertal et al., 2017). Whether and, if so, how spontaneous single cell/network rhythmicity affects AOB physiology and, consequently, AMC sensory coding remains unclear.

Throughout the nervous system, spontaneous activity, and rhythmic discharge in particular, is a major determinant of a neuron's coding capacity and information transfer function (Rieke et al., 1997; Buzsáki, 2006). At the population level, synchronized oscillatory activity tunes the temporal circuit dynamics (Buzsáki et al., 2013) and provides precise windows of excitability for circuit computations (Mizuseki et al., 2009). Neuronal oscillations span a broad frequency range from infra-slow (<0.1 Hz) to ultra-fast (200-600 Hz) frequencies, and changes in frequency bands often signify different physiological brain states or sensory processing (Buzsáki and Draguhn, 2004; Buzsáki, 2006). Within networks, synchronized rhythmic discharge can be controlled by neurons endowed with intrinsic pacemaker properties (Marder and Bucher, 2001; Cinelli et al., 2013). Alternatively, oscillations may emerge as a circuit property from selective synaptic wiring schemes and balanced periods of excitation and inhibition (Buzsáki, 2006; Fries, 2015). Infra-slow rhythms, which generate large, synchronous membrane potential fluctuations among cell assemblies (Steriade et al., 1993a,b), can reset and temporally bias local computation via phase and amplitude coupling (Buzsáki et al., 2013). Notably, the prolonged hyperpolarized “down” state during slow oscillations often results from the lack or extreme paucity of synaptic activity, rather than active inhibition (Buzsáki, 2006). In general, orchestrating periodic neuronal activity into synchronized cell assemblies bears attractive computational properties (Brody and Hopfield, 2003) and allows for an effective exchange of information among networks within a coordinated temporal reference structure (Destexhe and Sejnowski, 2003; Fries, 2015).

Here, we report infra-slow synchronous rhythmic activity of individual AOB glomerular units in awake freely behaving mice. We show that distinct ensembles of AMCs group into microcircuits that exhibit correlated discharge and, thus, underlie glomerular oscillations. Our results strongly suggest that infra-slow activity is driven by intrinsically rhythmogenic pacemaker-like neurons that entrain members of the same AMC local network motif via excitatory synaptic input.

## Materials and Methods

### 

#### Animals

All animal procedures were approved by local authorities at RWTH Aachen University and Boston University, were performed in accordance with local Animal Care and Use Committees' regulations, and were in compliance with European Union legislation (Directive 2010/63/EU) and recommendations by the Federation of European Laboratory Animal Science Associations. C57BL/6 mice (Charles River Laboratories) were housed in groups of both sexes (room temperature; 12:12 h light-dark cycle; food and water available *ad libitum*). All electrophysiological *in vitro* experiments used slices from young adults of either sex. We did not observe sex-dependent differences. For both *in vitro* and *in vivo* Ca^2+^ imaging experiments, the fluorescent Ca^2+^ indicators GCaMP6f (*in vitro* imaging) or GCaMP6s (*in vivo* imaging), respectively, were selectively expressed in olfactory bulb mitral and tufted cells (including AMCs) either by crossing Tbet-Cre mice (Haddad et al., 2013) to mice of the Ai95D reporter line (GCaMP6f; JAX stock #024105, The Jackson Laboratory) or by viral gene transfer in Tbet-Cre mice using AOB-targeted stereotaxic injection with conditional viral vectors (GCaMP6s; AAV9.DIO.GCaMP6s; UPenn Vector Core).

#### Chemicals and solutions

The following solutions (S_1_-S_6_) were used:

(S_1_) HEPES-buffered extracellular solution containing the following (in mM): 145 NaCl, 5 KCl, 1 CaCl_2_, 1 MgCl_2_, 10 HEPES, pH 7.3 (adjusted with NaOH), 300 mOsm (adjusted with glucose).

(S_2_) Oxygenated (95% O_2_/5% CO_2_) aCSF containing the following (in mM): 124 NaCl, 26 NaHCO_3_, 3 KCl, 1.25 NaH_2_PO_4_, 1.3 MgSO_4_, 1.3 CaCl_2_, 10 glucose, pH 7.3, 300 mOsm (adjusted with glucose).

(S_3_) Oxygenated (95% O_2_/5% CO_2_) cutting solution containing the following (in mM): 220 sucrose, 26 NaHCO_3_, 3 KCl, 1.25 NaH_2_PO_4_, 2.6 MgSO_4_, 10 glucose, pH 7.3, osmolarity = 300 mOsm (adjusted with glucose).

(S_4_) Elevated extracellular K^+^ solution containing the following (in mM): 100 NaCl, 50 KCl, 1 CaCl_2_, 1 MgSO_4_, 10 HEPES, pH 7.3, 300 mOsm (adjusted with glucose).

(S_5_) Standard pipette solution containing the following (in mM): 125 K-gluconate, 10 KCl, 2 KOH, 2 MgCl_2_, 1 EGTA, 0.3 CaCl_2_, 10 HEPES, 2 Mg-ATP, 1 Na-GTP (free Mg^2+^ = 2 mm; free Ca^2+^ = 130 nm), pH 7.1 (adjusted with KOH), osmolarity = 290 mOsm.

(S_6_) Symmetrical chloride pipette solution containing the following (in mM): 143 KCl, 2 KOH, 1 EGTA, 0.3 CaCl_2_, 10 HEPES, 2 Mg-ATP, 1 Na-GTP (free Ca^2+^ = 130 nm), pH 7.1 (adjusted with KOH); osmolarity = 290 mOsm.

Free Ca^2+^ concentrations were calculated using Ca-EGTA Calculator version 1.2 (https://somapp.ucdmc.ucdavis.edu/pharmacology/bers/maxchelator/CaEGTA-NIST.htm). If not stated otherwise, chemicals were purchased from Sigma Millipore. AlexaFluor hydrazide was purchased from Invitrogen; 2-(3-carboxypropyl)−3-amino-6-(4-methoxyphenyl)pyridazinium bromide (gabazine), AP5, and NBQX were purchased from Abcam. Final solvent concentrations were ≤0.1%. Solutions and pharmacological agents were applied either by bath or from air pressure-driven reservoirs via an 8-in-1 multibarrel “perfusion pencil” (Science Products). Changes in focal superfusion (Veitinger et al., 2011) were software-controlled and, if required, synchronized with data acquisition by transistor-transistor logic input to 12 V DC solenoid valves using a TIB 14S digital output trigger interface (HEKA Elektronik).

#### Slice preparation

Mice were killed by brief exposure to a CO_2_ atmosphere and decapitation. The left and right olfactory bulbs were rapidly removed while submerged in ice-cold oxygenated cutting solution (S_3_), then separated with a razor blade, and embedded in 4% low-gelling temperature agarose (VWR). Parasagittal slices (250 µm) were cut with a VT1000S vibratome (Leica Biosystems) in ice-cold S_3_. Two slices per bulb, each including the AOB, were transferred to a submerged, oxygenated storage container and allowed to recover for ≥1 h in aCSF (S_2_). Slices were then stored at room temperature until use.

#### *In vitro* electrophysiology

Olfactory bulb slices were transferred to a recording chamber (Luigs & Neumann), positioned with stainless-steel anchors, and visualized using an upright fixed-stage video-microscope (DM LSFA, DM6000FS or DM6FS, Leica Microsystems) equipped for infrared-optimized differential interference contrast. Slices were continuously superfused with oxygenated S_2_ (∼3 ml/min, gravity flow, 25°C). Neurons were visualized using a 5× (N Plan 5×/0.12) and 25× (HCX IRAPO L25×/0.95W) objective, a three-position magnification changer (0.35×, 1.25×, and 4.0×), and a cooled CCD camera (DFC360FX, Leica Microsystems). Patch pipettes (5-8 MΩ) were pulled from borosilicate glass capillaries (outer diameter, 1.50 mm; inner diameter, 0.86 mm; Science Products) on a PC-10 micropipette puller (Narishige Instruments), fire-polished (MF-830 Microforge, Narishige Instruments), and filled with pipette solution (S_5_ or S_6_, depending on experimental design). AlexaFluor-488 hydrazide (20 μm), and, in some recordings, biocytin [0.3% (w/v)] was added to the pipette solution to enable online evaluation of cell morphology and *post hoc* 3D reconstruction of recorded neurons, respectively. As demonstrated in previous recordings from AMCs (Gorin et al., 2016), neither chemical showed an evident effect on mitral cell electrophysiology. An agar bridge (150 mm KCl) connected the reference electrode and bath solution. EPC-10 USB amplifiers (single or double) controlled by Patchmaster 2.67-2.93 software (HEKA Elektronik) were used for data acquisition. To minimize electrical network noise, a 50/60 Hz noise eliminator (HumBug, Quest Scientific) was connected to the amplifier. We monitored and compensated pipette and membrane capacitance (C_mem_) as well as series resistance. Only neurons exhibiting relatively low (<30 MΩ) and stable access resistances were used for analysis. Liquid junction potentials were calculated using JPCalcW software (Barry, 1984) and corrected online. Signals were low-pass filtered [analog 3- and 4-pole Bessel filters (–3 dB); adjusted to one-third to one-fifth of the sampling rate (10 kHz)]. If not stated otherwise, holding potential (*V*_hold_) was –75 mV. All electrophysiological data were recorded at room temperature. Mitral cells were identified according to their location (residing in the external cellular layer between the AOB glomerular layer and the lateral olfactory tract) (Larriva-Sahd, 2008), soma size (large somata; C_mem_ ∼15 pF), and dendritic morphology (multiple apical/primary dendrites that terminate as tufts in the glomerular layer). Action potential-driven capacitive currents were recorded from intact mitral cell somata in loose-seal cell-attached configuration (seal resistance 30-150 MΩ; pipettes filled with S_1_) to prevent dialysis of intracellular components. Passive membrane properties [i.e., input resistance (R_input_), C_mem_, and membrane time constant (τ_mem_)] were obtained immediately after membrane rupture. Treated, to a first approximation, as a “biological constant” with a value of ∼1 μF/cm^2^ (Gentet et al., 2000), C_mem_ was determined using a square pulse (5 mV, 10 ms) routine. R_input_ at the mitral cell soma was determined by measuring the steady-state voltage response to a hyperpolarizing current step of −70 pA. Linear passive voltage responses were also used to estimate τ_mem_ from monoexponential fits to the voltage responses (from onset to steady state).

#### Ca^2+^ imaging

*In vitro* imaging of AMC activity in acute slices was performed in a recording chamber (Luigs & Neumann) mounted on an upright fixed-stage scanning confocal microscope (TCS SP5 DM6000CFS, Leica Microsystems) equipped with a 20×/1.0 NA water immersion objective (HCX APO L, Leica Microsystems), infrared-optimized differential interference contrast optics, and a cooled CCD-camera (DFC360FX, Leica Microsystems). Slices were continuously superfused with oxygenated S_2_ (∼5 ml/min; gravity flow). GCaMP6f was excited at 488 nm (multiline argon laser; <25% laser power), and fluorescence was detected within a 500-600 nm spectral band. Changes in cytosolic Ca^2+^ were monitored at 1.0 Hz frame rate (1024 × 512 pixels; 400 Hz bidirectional scanning frequency) using LAS X software (Leica Microsystems). Pinhole adjustment restricted optical *z*-section size to 5-10 µm. Recording duration for each experimental condition was ≥10 min.

For *in vivo* imaging, Tbet-Cre mice (Haddad et al., 2013) were anesthetized with isoflurane (1.5%) and a small craniotomy was opened over the olfactory bulb. For selective expression in AMCs, the AOB was targeted using stereotaxic coordinates and injected with conditional viral vectors encoding the Ca^2+^ indicator GCaMP6s (∼50-150 μl of virus solution; titer 10^12^/µl diluted 4× in sterile cortex buffer). After allowing ∼21 d for expression, mice were again anesthetized and a craniotomy was opened 1.5-2.0 mm posterior to the transverse sinus separating the olfactory bulbs and frontal cortex. A small cylinder of cortex (1 mm diameter) was aspirated to expose the rear face of the main and AOBs, and a 1-mm-diameter/4-mm-length gradient-index (GRIN) lens was inserted abutting the tissue surface. The GRIN lens was fixed with silicone sealant (Kwik-Sil) and cemented with dental acrylic (Metabond, Parkell). After 2-3 weeks of recovery, mice were briefly anesthetized to attach a miniaturized head-mounted fluorescence microscope, or “mini-scope” (Liberti et al., 2016, 2017), which captured fluorescence signals relayed from the AOB by the GRIN lens. Placement over the AOB rather than MOB was confirmed by lack of respiratory-coupled activity, lack of fluorescence increases driven by volatile odorants, and, in some cases, by histologic evaluation after imaging was complete.

*In vivo* data were collected using previously described custom hardware and acquisition code (Liberti et al., 2016, 2017). Mice were placed in a clean acrylic arena to minimize external sensory input. In the absence of conspecifics or external cues, mice typically became quiescent after an initial investigatory period. For comparison with oscillations observed in slices, imaging was performed during periods of rest when the AOB was least subject to sensory input or state-dependent modulation. Image series were collected at 10-30 Hz for periods of 3-10 min, depending on the time spent in a quiescent state.

#### Experimental design and statistical analysis

All *in vitro* data were obtained from independent experiments performed on ≥3 d using ≥3 different animals. Individual numbers of cells/experiments (*n*) are denoted in the figures and/or legends. If not stated otherwise, results are presented as mean ± SEM. Statistical analyses were performed using paired or unpaired *t* tests, one-way ANOVA with Tukey's HSD *post hoc* test, Wilcoxon rank sum tests, Wilcoxon signed rank tests, Mann–Whitney *U* test with Bonferroni correction for multiple comparisons, or Fisher-*z* transformation (as dictated by data distribution and experimental design). Tests and corresponding *p* values that report statistical significance (≤ 0.05) are individually specified in figure legends. Data were analyzed offline using IGOR Pro 6.3-8.0 (WaveMetrics), MATLAB 2018 (The MathWorks), and Excel 2016 (Microsoft) software. Time constants (τ) were calculated by fitting individual traces to monoexponential functions *I*_(t)_ = *I*_1_ [exp (- *t/*τ)] + *I*_0_. Synaptic currents in continuous whole-cell patch-clamp recordings were analyzed using IGOR Pro functions (SpAcAn: Spontaneous Activity Analysis, written by Guillaume Dugué and Charly Rousseau) for detection and analysis of spontaneous events by a threshold detection algorithm.

Images from *in vitro* experiments were registered using either ImageJ 1.51n (rigid body correction) or spyder 3.1.2 (SIMA motion correction) (Dombeck et al., 2007) depending on drift direction. AMC somata were delimited as ROIs. To correct for neuropil contamination, the signal intensities of additional ROIs surrounding each soma were subtracted from the somatic signal offline. For each ROI, the fluorescence intensity and center of mass were calculated using ImageJ 1.51n. For classification of AMC activity (ir)regularity, we used custom-written scripts in MATLAB to determine each neuron's auto-correlation (*xcorr* function) and power spectral density (PSD; *pwelch* function). We classified AMCs as oscillating if activity analyses met two criteria in the time and frequency domains: (1) auto-correlograms displayed clearly discernible side peaks and two pronounced negative troughs flanking the peak at zero lag; and (2) PSD plots showed at least one clear peak within the 0.01-0.3 Hz frequency range. Depending on raw data signal strength, PSD threshold was set to either 3.5 AU (raw peak signals <7) or 10 AU (all raw peak signals >7). To account for variable signal strength that might result from neuron-to-neuron differences in optical section diameter, GCaMP6f expression level, etc., we adjusted peak detection thresholds to raw Ca^2+^ signal intensities.

To determine synchronous activity among AMC ensembles, all simultaneously recorded neurons that were classified as oscillating were then subjected to pairwise cross-correlation analysis. For each AMC pair, we calculated the peak cross-correlation coefficient (*corrcoef* function, 5 min sliding windows, 1 min shifts; MATLAB) allowing ±5 s lag. To identify significant correlations, we plotted cross-correlation coefficient histograms (including coefficients from all AMC pairs within each 5 min window; *n* = 178,664; control condition). By fitting a Gaussian function to the histogram's left slope and peak, we calculated a threshold value corresponding to the 95th percentile point of this normal distribution. All AMC pairs that showed cross-correlation coefficients exceeding this threshold were classified as significantly correlated. Next, for each experiment, correlated/synchronous (±5 s lag) activity among AMC ensembles was identified by cluster analysis. Individual clusters fulfilled the following constraints: (1) all AMC pairs within a cluster were significantly correlated; and (2) while individual AMCs can sometimes be part of multiple unique clusters, smaller AMC subsets within an ensemble do not constitute bona-fide clusters.

*In vivo* imaging time series of AOB GCaMP6s signals contained both focal components, presumably corresponding to glomerular activity, as well as a diffuse global component resulting from scattered fluorescence from deeper somata and dendrites. In some cases, signals met our criteria for AOB attribution, but we were unable to focus on the glomerular layer. In such cases, we monitored the average signal intensity over the entire imaging field, providing integrated quasi “fiber photometry” data from large AMC populations. Fluorescence time-lapse recordings were high-pass filtered, removing components <0.03 Hz to correct for slow temperature-dependent drift in LED intensity. Auto-correlations and PSDs were calculated in MATLAB (*autocorr* and *pwelch* functions).

When images contained both global and focal components, glomerular signals were isolated by subtracting a low-pass-filtered version of the image series, calculated by convolving with a Gaussian kernel (30 µm width) (Meister and Bonhoeffer, 2001). The resulting high-pass-filtered data were motion-corrected using rigid-body translation (Turboreg plugin; ImageJ). For fluorescent intensity analysis, we defined ROIs based on the original time-lapse recording as well as both maximum intensity and SD projections of the image series. As ΔF/F values are affected by high-pass filtering, all analyses were based on mean pixel intensities from each ROI. Power spectra of, and cross-correlations between, glomerular ROIs were computed in MATLAB. Significant correlations between glomerular activity were assessed using a bootstrap method, where pairwise cross-correlations were computed after shifting the time series for each individual ROI by a random amount. Repeating this process 10,000 times generated a shuffled distribution. Significance was assigned to glomerular pairs using the upper and lower 5% bounds of this shuffled distribution. For cluster analysis, similarity trees were constructed in MATLAB (*linkage* function) based on average correlation distance. Correlated glomeruli were grouped (*cluster* function) with the maximum number of detected clusters limited to a value between 8 and 20. To test whether activity correlations between glomeruli showed any spatial dependence, we calculated both the distance and Pearson correlation for all possible pairwise comparisons over a distance range of 0-600 µm. Next, we calculated the mean correlation coefficient for all pairs falling within 50 µm bins. Shuffled distributions used for bootstrap comparisons were calculated using the same approach, after randomly reassigning correlation-distance values for all pairs and repeating 10,000 times. We compared the measured and shuffled distributions for each distance range using a Mann–Whitney *U* test with Bonferroni correction for multiple comparisons.

To address whether synchronous activity was stable throughout the imaging period, we divided each session into two equal periods, and compared Pearson correlation coefficients for the intensity time series for the first and second halves of the session.

## Results

Recently, we and others (Gorin et al., 2016; Vargas-Barroso et al., 2016; Zylbertal et al., 2017) reported that slow to infra-slow oscillations with remarkable periodicity represent the default activity pattern of a subset of AMCs *in vitro*. Whether such rhythmic activity exists in awake animals and, if so, whether these activity patterns reflect any degree of functional organization of the AOB circuitry is unknown.

### Infra-slow rhythmic activity on a glomerular scale represents the idle state of AOB output in awake mice

**Figure 1.**
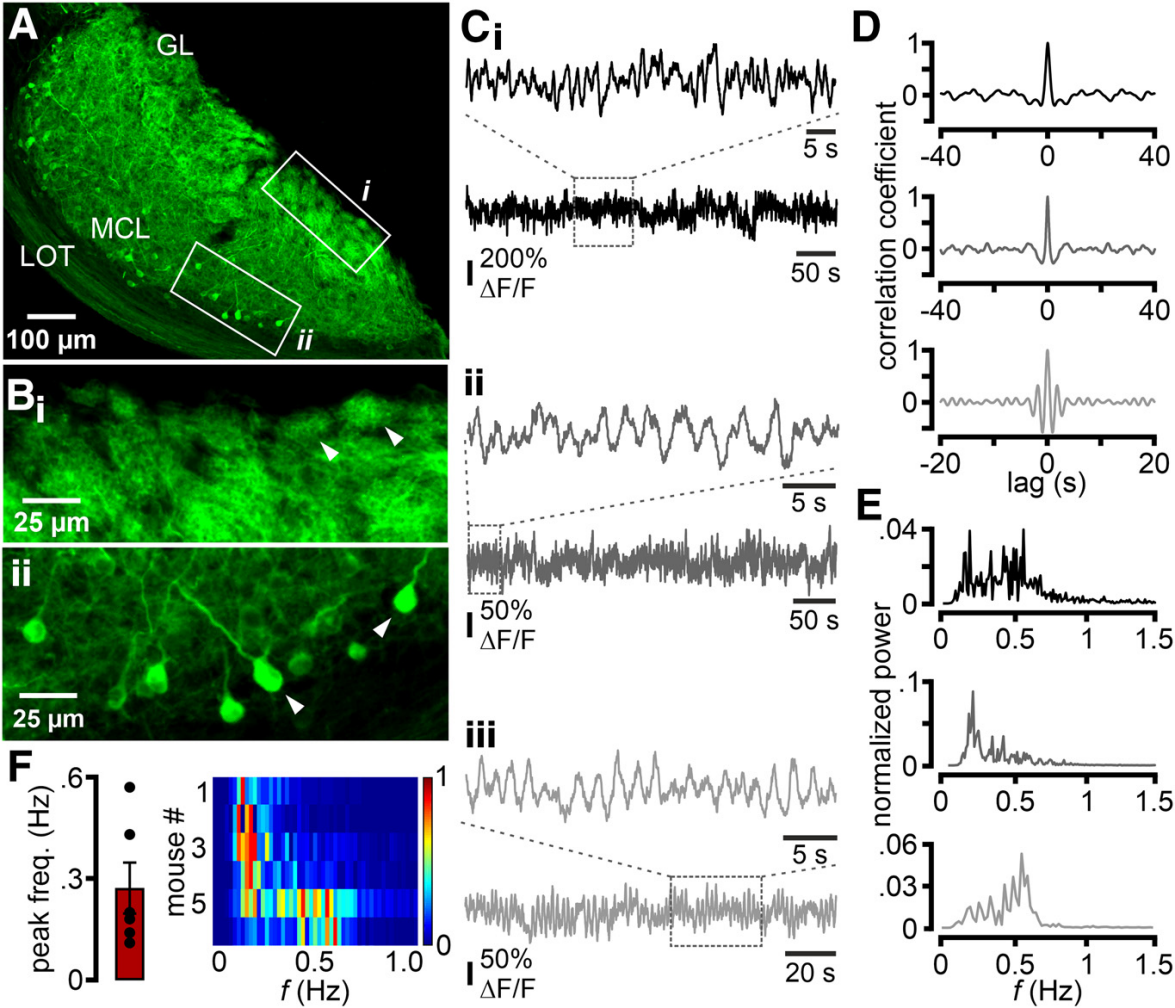
Slow rhythmic Ca^2+^ fluctuations in the resting-state AOB of awake mice. ***A***, Hemi-sagittal section illustrates conditional GCaMP6s expression in AMCs of Tbet-Cre mice after viral gene transfer. GL, Glomerular layer; LOT, lateral olfactory tract; MCL, mitral cell layer. ***B***, Enlarged view of boxed areas in ***A*** demonstrating GCaMP6s expression in both apical dendrites within the glomerular layer (***B_i_***) and AMC somata in deeper layers (***B_ii_***). Arrowheads indicate individual glomeruli (***B_i_***) and somata (***B_ii_***), respectively. ***C***, Representative original traces of the average integrated (“bulk”) GCaMP6s signal intensity (ΔF/F) recorded from the AOB of 3 different animals (***C_i_–C_iii_***) during periods (>3 min) of behavioral quiescence. Dashed rectangles represent segments that are shown on extended time scales. Examples reveal periodicity at frequencies <1 Hz (frequencies <0.03 Hz were filtered to correct for drift in illumination). ***D***, ***E***, Temporal and frequency analysis of the example signals shown in ***C***. Auto-correlograms (***D***) and power spectra (***E***) reveal signal periodicity. Note the occurrence of several prominent peaks at <1 Hz. ***F***, Power spectra (heat map) and peak frequencies (0.27 ± 0.08 Hz; mean ± SEM) of integrated AOB activity in 6 animals.

Initially, we asked whether rhythmic neural activity manifests in the AOB of awake, unrestrained animals. To address this, we selectively expressed the genetically encoded Ca^2+^ indicator GCaMP6s (Chen et al., 2013) in the AOB of Tbet-Cre mice (Haddad et al., 2013) by stereotaxic adeno-associated virus injection. Robust and selective GCaMP6s expression in AMCs, including both somata and dendritic tufts in the glomerular layer, was observed by *post hoc* histology (Fig. 1*A*,*B*). We then recorded *in vivo* Ca^2+^ dynamics within AMC apical dendrites in the AOB glomerular layer neuropil, using a head-mounted miniature microscope (Liberti et al., 2016, 2017) attached to an implanted GRIN relay lens that targeted the AOB from the rear of the animal. In six mice separately placed in a clean circular arena, we monitored the average “bulk” Ca^2+^ signal intensity within the entire glomerular imaging field over prolonged periods of behavioral quiescence (≤10 min; Fig. 1*C*), reflecting collective AMC activity. Notably, signals displayed continuous periodic intensity fluctuations (Fig. 1*C*,*D*). Spectral density analysis revealed several distinct peaks at different frequencies within the power spectrum (Fig. 1*E*). These multiple bands were primarily concentrated to <1 Hz. Dominant frequencies in individual animals ranged between 0.14 and 0.60 Hz (0.27 ± 0.08 Hz; mean ± SEM; Fig. 1*F*), although significant power remained in bands up to ∼0.7 Hz. These data demonstrate substantial AOB resting activity in awake, but inactive animals. The observed low-frequency bands of strong periodicity bear striking spectral resemblance to oscillations previously described in individual AMCs *in vitro* (Gorin et al., 2016; Vargas-Barroso et al., 2016; Zylbertal et al., 2017).

**Figure 2.**
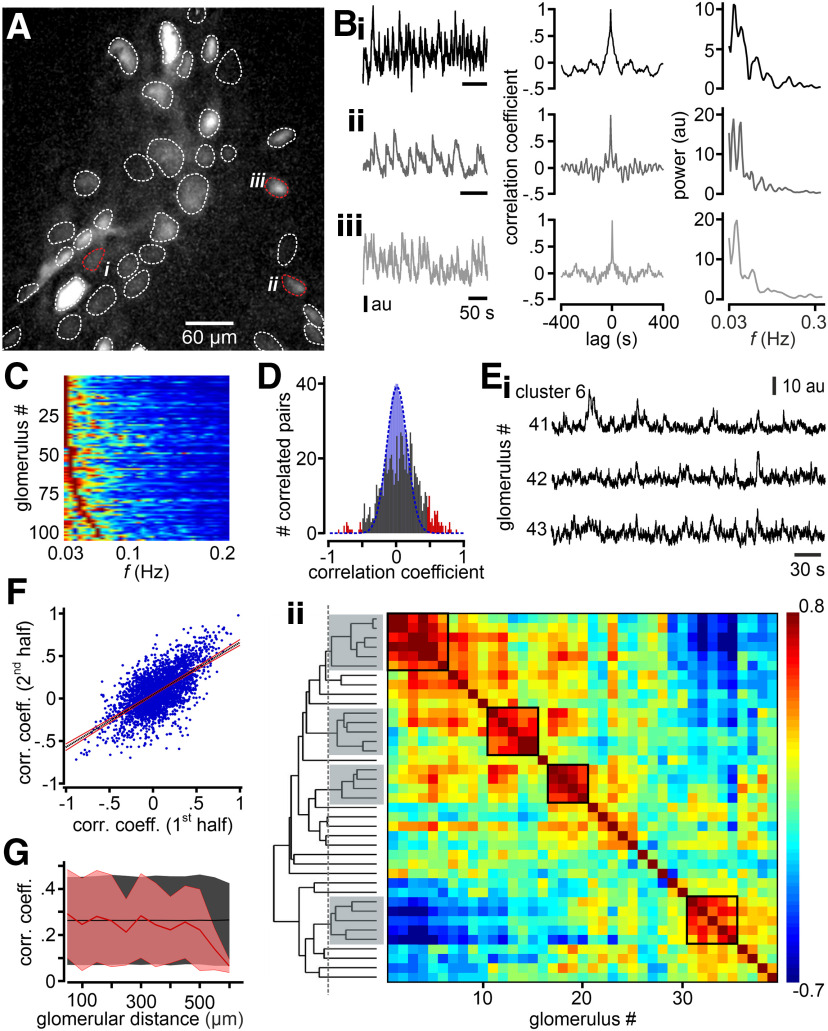
Rhythmic activity in single AOB glomeruli and correlated oscillations across subsets of glomeruli *in vivo*. ***A***, Individual glomeruli, marked as ROIs (dotted lines), which show spontaneous activity during periods of behavioral quiescence, are identified by an SD projection of GCaMP6s fluorescence over the image time series. ***B_i_–_iii_***, Average GCaMP6s signal intensity in arbitrary units (au) from three representative glomeruli (***A_i_*–*A_iii_***, red dotted lines) over time. Original traces (left), auto-correlograms (middle), and power spectra (right) reveal periodic glomerular activity on multiple time scales within the infra-slow frequency range (<0.3 Hz). ***C***, Heat map represents spectral power for all glomeruli investigated. ***D***, Histogram of pairwise correlation coefficients calculated for all glomerular pairs shown in ***A***. Based on comparison with a random time-shuffled distribution (blue Gaussian curve), bootstrap analysis identifies significantly correlated pairs of glomeruli (red). ***E***, Hierarchical clustering analysis reveals subsets of correlated glomeruli that are apparent from both original recordings (***E_i_***) and cross-correlation matrices (***E_ii_***). ***E_ii_***, Dashed vertical line indicates the similarity threshold for cluster assignment. ***F***, Correlated glomerular activity remains stable throughout the imaging period. Scatter plots of Pearson correlations for the first *versus* second half of the recording session. Each blue dot represents one pairwise correlation. Black dashed line indicates a linear fit with 99% CIs (red lines). ***G***, Pairwise correlations between glomeruli as a function of distance. Each point represents the mean Pearson correlation coefficient (±SD; shading) for all glomerular pairs falling within 50 µm distance bins. Red line and shading represent measured distribution. Black line and shading represent shuffled distribution. Pooled data showed no significant differences between measured and shuffled values at any distance range (Mann–Whitney *U* test with Bonferroni correction), which is consistent with a generally random spatial organization.

We next asked how oscillatory activity is distributed across AMC subpopulations. When images showed spatially distinct foci of activity, presumably corresponding to AMC dendrites compartmentalized within different glomeruli, we extracted intensity time series for each of these foci in the imaging field (Fig. 2*A*). Notably, oscillations were also apparent at the glomerular scale (Fig. 2*B*). Auto-correlation analysis of single-glomerulus signals revealed diverse and more pronounced periodicity than seen in bulk AOB recordings (Fig. 1*D*). Moreover, spectral power was confined to one or few peaks in a concentrated frequency range between 0.03 and 0.5 Hz, most of them clustered at the lower end of this range (Fig. 2*B*,*C*). Together, these data show that individual glomeruli display a characteristic rhythmicity, suggesting that bulk AOB signals contain contributions from multiple AMC populations with disparate temporal characteristics.

Since AMCs target multiple glomeruli (Takami and Graziadei, 1991; Urban and Castro, 2005; Yonekura and Yokoi, 2008), we next investigated whether oscillatory activity is synchronized among subsets of glomeruli or is, instead, independent across foci of activity. Based on the individual intensity time series for each glomerulus, we constructed cross-correlograms from each glomerular pair (two animals). To identify glomerular pairs with statistically significant cross-correlation, we compared the distribution of Pearson correlation coefficients from all measured pairs with those for shuffled data (Fig. 2*D*; see Materials and Methods). In addition, hierarchical clustering revealed subsets of significantly correlated glomeruli (Fig. 2*Ei*) that also become apparent in experiment-specific cross-correlation matrices (Fig. 2*Eii*). In total, our experiments revealed 10 clusters of ≥3 correlated glomeruli. Next, we examined thedynamics of correlated glomerular activity. Comparison of individual pairwise correlation coefficients between the first and the second half of the recording period revealed that, in the idle state, correlations between glomeruli are generally stable (Fig. 2*F*). Finally, we asked whether correlated glomeruli showed any nonrandom spatial distribution. We calculated the mean pairwise correlation coefficients for all glomerular pairs within bins of 50 µm distances and plotted these as a function of distance (0-600 µm). Comparing the resulting distribution to shuffled data (Fig. 2*G*; see Materials and Methods), we found no evidence for spatial clustering.

Together, Ca^2+^ imaging of AMC activity in the glomerular layer of awake mice indicates that, at rest, the AOB displays glomerular patterns of significant oscillatory activity with strikingly slow periodicity. The exact correspondence between glomerular signals and somatic AMC firing is unclear. However, since backpropagating somatic action potentials elicit strong Ca^2+^ transients in AMC dendrites and their glomerular tufts (Ma and Lowe, 2004), our data strongly suggest that these oscillations emerge from synchronized activity among selected AMC ensembles.

### AMCs assemble into functional ensembles that exhibit correlated periodic activity

**Figure 3.**
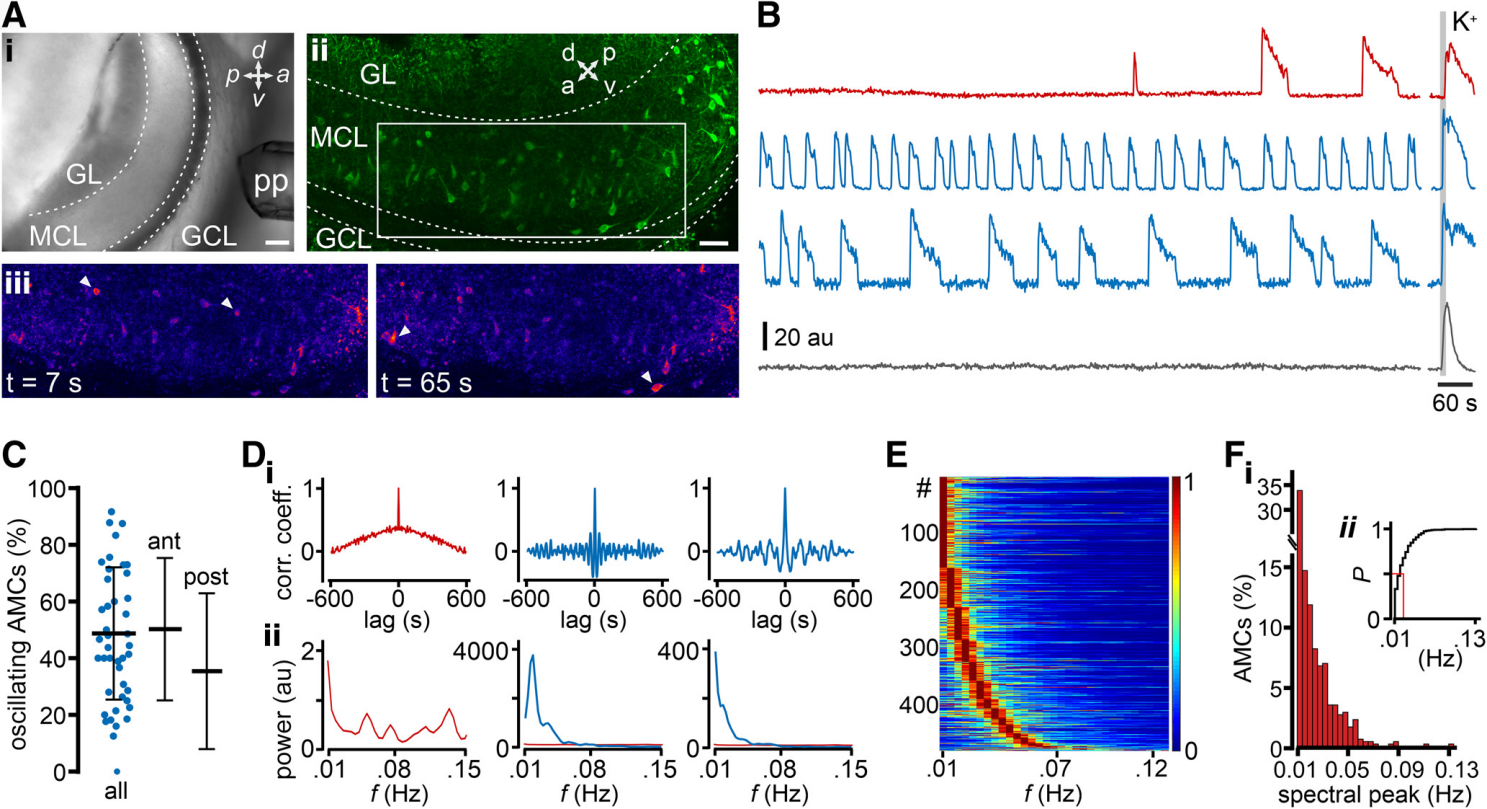
AMC population imaging in acute slices reveals diverse patterns of spontaneous activity. ***A***, Experimental setup for confocal population Ca^2+^ imaging in acute AOB slices. ***A_i_***, Differential interference contrast overview of a sagittal section shows the layered structure of the AOB and the position of the perfusion pencil (pp). Scale bar, 100 µm. a, Anterior; d, dorsal; p, posterior; v, ventral. ***Aii***, Pseudocolor (green) 20-frame maximum projection of GCaMP6f fluorescence in the AOB of a Tbet-Cre x Ai95D mouse. Individual AMC somata are clearly discernible. Scale bar, 50 µm. GCL, Granule cell layer; GL, glomerular layer; MCL, mitral cell layer. ***A_iii_***, Two single frames (boxed area in ***A_ii_***) recorded at different time points indicate transient activity in different neurons (arrowheads). ***B***, Representative original recordings of average fluorescence intensity (au, arbitrary units) from four different AMCs as a function of time. Traces represent either irregular (red) or periodic (blue) bursts of activity, respectively, or silent neurons (black). Depolarization-dependent signals evoked by elevated extracellular K^+^ (50 mm) serve as viability controls. ***C***, Dot plot represents the fraction of oscillating AMCs per experiment. On average, 48.7 ± 23.3% (mean ± SD) of AMCs display oscillatory Ca^2+^ signals. Similar fractions are found in the anterior (ant; 50.4 ± 25.1%) and posterior (post; 35.6 ± 27.5%) parts of the AOB. Note the large variability that is independent of spatial distribution. ***D***, Auto-correlograms (***D_i_***) and power spectra (***D_ii_***) constructed from the red and the two blue traces shown in ***B***. For comparison, the power spectrum of the irregularly bursting AMC (red trace in ***B***) is shown in all three PSD plots. ***E***, Heat map represents normalized power spectra for a total of 494 AMCs, sorted according to peak frequency band. ***F_i_***, Histogram represents the distribution of spectral power peaks within the population of oscillating AMCs. ***F_ii_***, Inset, Cumulative probability (red line indicates *P*_0.5_ = 0.016 Hz) of frequencies with peak power.

**Figure 4.**
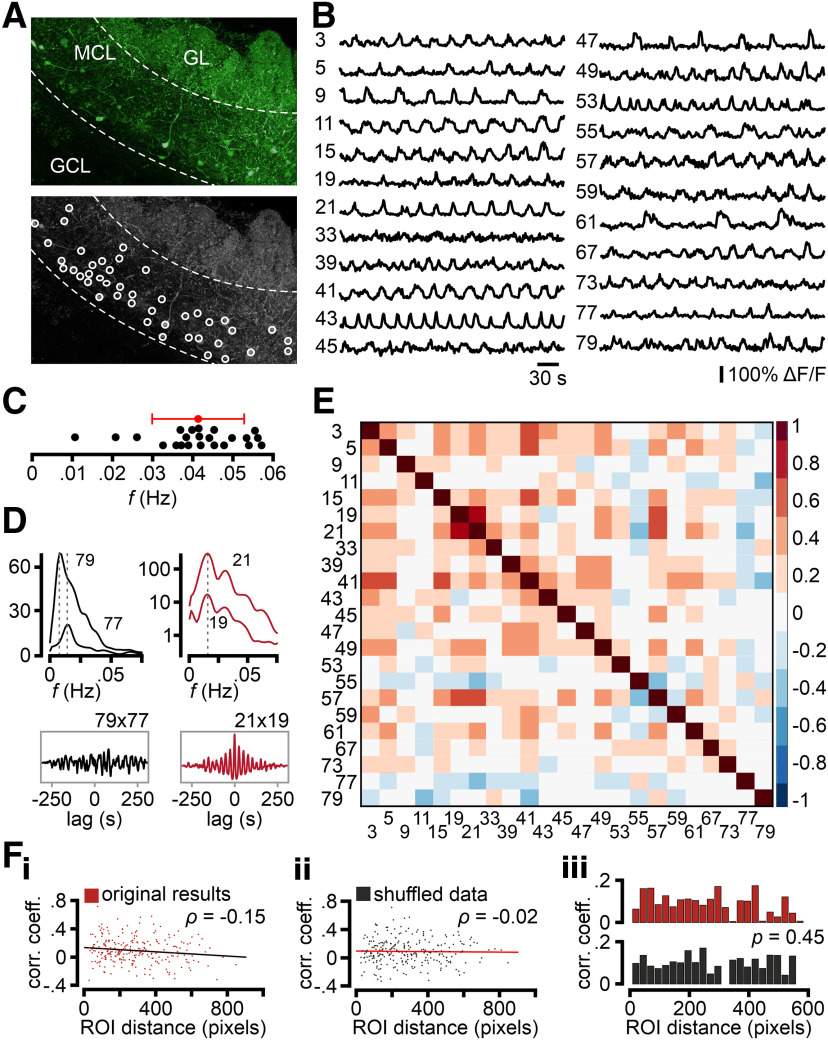
Considerable variability in AMC oscillation frequencies. ***A***, Pseudocolor (top; green) maximum projection of GCaMP6f fluorescence in an acute parasagittal AOB section from a Tbet-Cre x Ai95D mouse. Oscillating AMCs (white ROIs; *n* = 23) are indicated in grayscale maximum projection (bottom). ***B***, Traces from individual oscillatory AMCs represent rhythmic variability between neurons. ***C***, Scatter dot plot represents the distribution of peak oscillation frequencies in single neurons (black dots). Data span a range between 0.01 and 0.06 Hz, with an average of 0.041 ± 0.011 Hz (red; mean ± SD). This variability highlights the lack of a single dominant frequency within a given slice. ***D***, PSD overlays (top) and cross-correlograms (bottom) of example pairs of uncorrelated (black, left) and correlated (red, right) AMCs. Corresponding ROI numbers as indicated. Dashed vertical lines (gray) in PSDs indicate maxima for each ROI. ***E***, Cross-correlation matrix represents pairwise analysis of zero-lag covariance for all oscillating signals. Positive/negative correlation coefficients are color-coded (blue-to-red look-up table; 0.2 bin width). ***F***, Pairwise signal correlation analysis (zero-lag covariance) plotted against physical 2D distance between AMC pairs. Measurement data (***F_i_***; original results) and randomly assigned pairs (***F_ii_***; shuffled data) are shown as scatter plots. Linear regression indicates that the two variables are not correlated [Pearson correlation coefficients, ρ = −0.15 (***F_i_***) and ρ = −0.02 (***F_ii_***), respectively]. When both original and shuffled data distributions are plotted as histograms (***F_iii_***; 25 pixel bin size), no spatial organization between correlated AMC pairs becomes evident (*p* = 0.45; Fisher-*z* transformation).

To investigate whether correlated AMC ensemble activity underlies glomerular oscillations, we performed confocal time-lapse Ca^2+^ imaging of large populations of GCaMP6f-expressing AMCs in acute sagittal AOB slices (Tbet-Cre x Ai95D mice; Fig. 3*A*). When monitoring AMC Ca^2+^ concentration for up to 33 min, the vast majority of neurons generated spontaneous signals (Fig. 3*B*). Corroborating our own previous electrophysiological findings (Gorin et al., 2016), spontaneously active AMCs displayed one of two distinct activity patterns: either irregular bursting with no apparent periodicity or infra-slow oscillations of variable temporal characteristics. On average, oscillating neurons made up ∼50% of all AMCs, although this proportion varied substantially across experiments (Fig. 3*C*). We did not observe any difference in oscillation probability according to AMC distribution along the anterior-to-posterior axis of the AOB. Categorization into (non)oscillatory populations was based on periodicity analysis in both the temporal and spectral domains (Fig. 3*D*). Power spectra of oscillating AMCs typically showed a single peak in a frequency range of 0.01-0.15 Hz. Similar to the spectral data obtained from individual glomeruli *in vivo* (Fig. 2*B*,*C*), AMC frequency peaks usually clustered to <0.05 Hz (Fig. 3*E*,*F*). Within this spectral range, however, peak distribution varied considerably among AMCs, both across and within experiments (Figs. 3*F*, 4*A–C*). Cross-correlation analysis of all AMC pairs (within a given experiment) revealed several pairs with high Pearson correlation coefficients, whereas others showed no apparent synchronization (Fig. 4*D*,*E*). Notably, the degree of signal synchronization is essentially unrelated to the distance between AMC pairs within the slice confocal plane, suggesting that correlated activity does not depend on spatial proximity (Fig. 4*F*).

**Figure 5.**
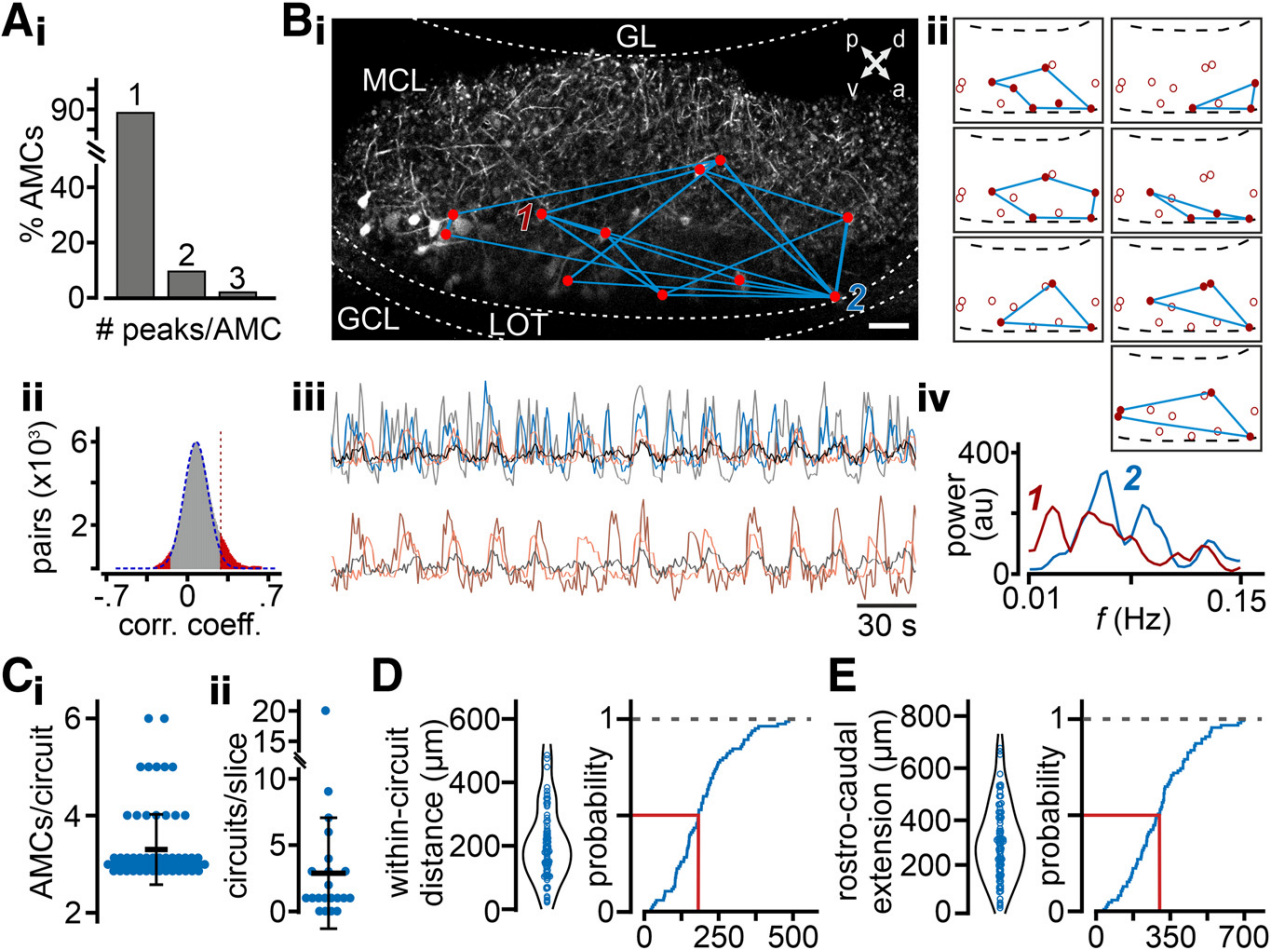
AMC subsets form synchronized oscillatory microcircuits. ***A***, Histograms represent the number of distinct power spectral peaks per AMC (***A_i_***), and the distribution of pairwise correlation coefficients (±5 s lag) calculated for all oscillating AMC pairs (***A_ii_***; *n* = 5763; each pair analyzed during 31 “sliding” 5 min windows; *n*_total_ = 178,664). Fitting a Gaussian function (dashed blue line) to the histogram's left slope and peak identifies significantly correlated pairs of glomeruli (red; threshold corresponding to the 95th percentile point of this normal distribution). ***B***, Grayscale maximum projection (***B_i_***) of GCaMP6f fluorescence (Tbet-Cre x Ai95D mouse) in an acute slice that contains seven synchronized AMC ensembles. Scale bar, 50 µm. GCL, Granule cell layer; GL, glomerular layer; LOT, lateral olfactory tract; MCL, mitral cell layer. Oscillating neurons (red dots) that showed significant correlation with two or more other AMCs are assigned to a microcircuit. Each of these is outlined (***B_i_***) and mapped (***B_ii_***) by blue connecting lines. ***B_iii_***, Original traces of three and five significantly correlated AMCs, respectively, illustrate synchronized activity within two of the seven circuits (corresponding to the top two maps in ***B_ii_***). While the vast majority of AMCs display one distinct peak in the PSD (***A_i_***), two AMCs (indicated as 1, red; and 2, blue; in ***B_i_***) that belong to several microcircuits display multipeak PSDs (***B_iv_***). ***C***, Microcircuits contain up to six neurons (***C_i_***) within a confocal optical section, and up to 20 individual ensembles (***C_ii_***) are found per slice (2.9 ± 4.2). ***D***, ***E***, Violin dot plots and cumulative probability plots quantify spatial microcircuit distribution within AOB slices. Analysis parameters are the mean within-circuit distance of AMC pairs (***D***; 195.6 ± 101.5 µm) and the maximum length along each circuit's rostrocaudal axis (***E***; 289 ± 150.5 µm). Red lines indicate *P*_0.5_ probabilities (182.5 µm, ***D***; 278.8 µm, ***E***).

Definition of statistically significant cross-correlations among AMC pairs (Fig. 5*Aii*; based on the distribution of Pearson correlation coefficients from all measured pairs as well as a stringent threshold criterion; see Materials and Methods) allowed identification of synchronized AMC ensemble activity (Fig. 5*B*). Within a given ensemble (microcircuit; defined as ≥3 synchronized neurons), all AMC pairs were significantly correlated. In a few cases, individual AMCs were part of multiple unique microcircuits (Fig. 5*Bi–Biii*). Notably, these neurons typically displayed multiple PSD peaks (Fig. 5*Ai*,*Biv*). Within the restricted spatial extent of a confocal optical *z* section, we found that up to six AMCs constituted a given microcircuit (Fig. 5*Ci*); and typically, we observed at least one synchronized ensemble per slice (Fig. 5*Cii*). Within the volume of the AOB, which harbors ∼7000 AMCs (Mohrhardt et al., 2018), *de facto* numbers must be considerably higher. While most microcircuits encompass a “two-dimensional” area of <10^4^ µm^2^ (data not shown), both pairwise AMC distance within a circuit and its rostrocaudal dimension appear homogeneously distributed across the extent of the AOB (Fig. 5*D,E*).

Together, confocal time-lapse imaging experiments reveal that groups of infra-slow oscillating AMCs assemble into distinct microcircuits that exhibit correlated Ca^2+^ signals, consistent with glomerular synchrony seen in *in vivo* data. Members of such ensembles are not spatially clustered along the AOB rostrocaudal axis. We conclude that AMC microcircuits with synchronized periodic activity reflect the idle state glomerular oscillations observed *in vivo*.

### Microcircuits contain both intrinsically rhythmogenic neurons and neurons that are synaptically entrained by network activity

**Figure 6.**
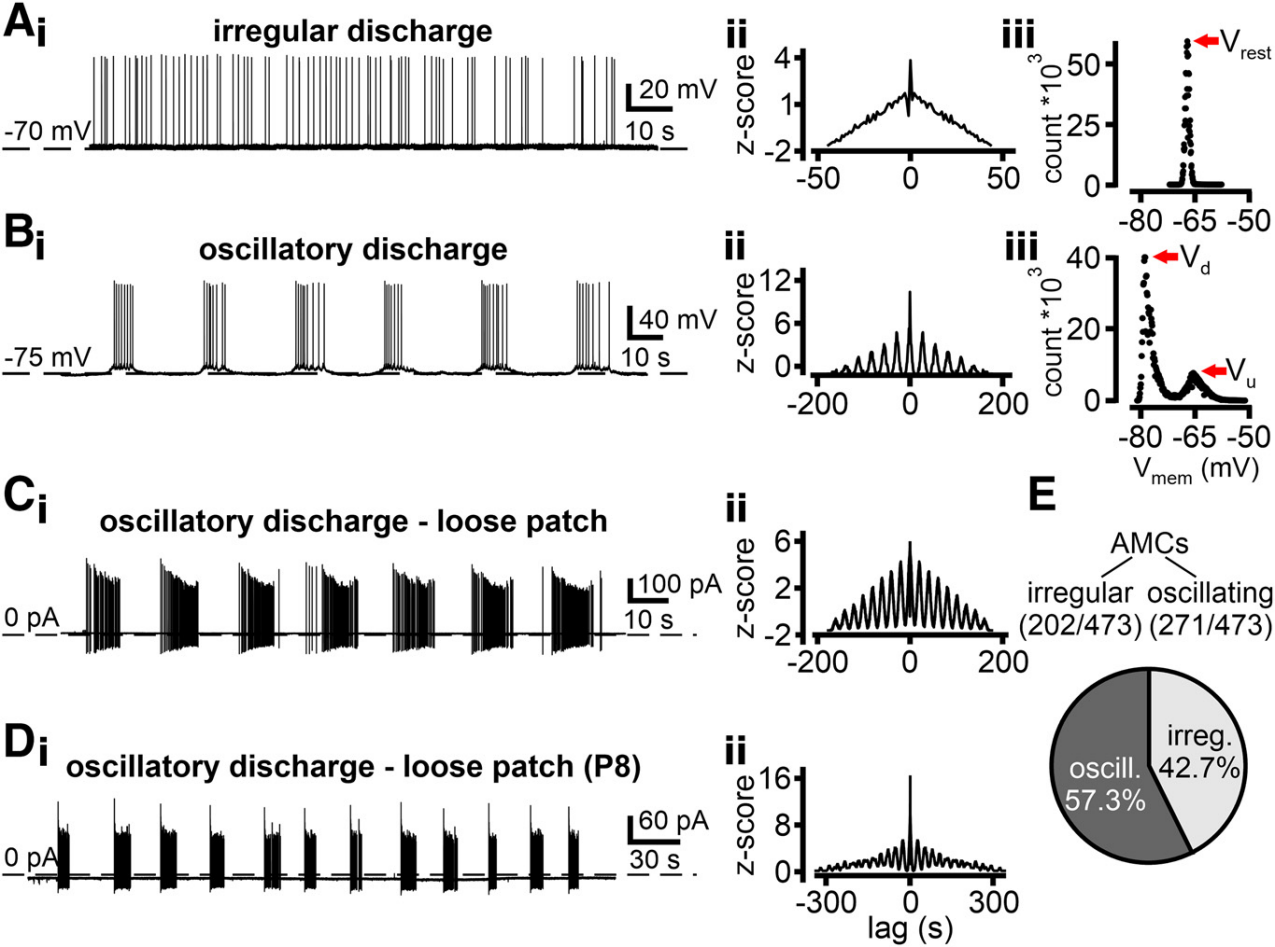
Electrophysiological single-neuron recordings reveal different patterns of spontaneous AMC activity *in vitro*. ***A***, ***B***, Representative whole-cell current clamp recordings of two distinct types of spontaneous discharge found in AMCs: mitral cells either spike irregularly (***A_i_***) or exhibit periodic discharge patterns (***B_i_***). Rhythmicity of action potential discharge (or the lack thereof) is evident in the corresponding auto-correlation histograms (***A_ii_***, ***B_ii_***; 1 s bin width). While irregularly firing AMCs exhibit a stable baseline membrane potential (V_rest_), reflected in a single peak in an all-points V_mem_ histogram (***A_iii_***; red arrow; 82 μV bin width), periodically discharging AMCs alternate between two membrane potentials. Membrane bistability of rhythmic AMCs is reflected by two distinct peaks in the all-points histogram (***B_iii_***; red arrows; 122 μV bin width) that correspond to a relatively hyperpolarized “down” state voltage (V_d_) and a more depolarized “up” state membrane potential (V_u_). ***C***, ***D***, Periodic bursting is also observed in extracellular loose-seal recordings (***i***) and corresponding auto-correlation histograms (***ii***) from AMCs in both adult (***C***) and juvenile mice as young as P8 (***D***). ***E***, Irregular spontaneous activity was found in 42.7% (202 of 473) of AMCs, whereas 57.3% (271 of 473) displayed oscillatory discharge.

Previously, we reported that the mouse AOB contains a group of intrinsically rhythmogenic AMCs that generate infra-slow membrane potential (V_mem_) oscillations independent of fast synaptic input (Gorin et al., 2016). To corroborate our previous findings, we initially recorded spontaneous electrical activity from individual AMCs in sagittal AOB sections. When continuously monitoring V_mem_ for prolonged periods of time under control conditions (0 pA current injection), AMCs displayed either irregular discharge (Fig. 6*A*) or periodic burst firing with recurring “up” and “down” states in the underlying subthreshold membrane potential (Fig. 6*B*). Essentially, the same spontaneous activity patterns were observed when we recorded AMC activity in “loose-seal” cell-attached configuration (Fig. 6*C*) to prevent dialysis of cytosolic components and maintain unperturbed resting membrane potentials. Notably, AMC oscillatory discharge was already apparent in recordings from neonatal animals (Fig. 6*D*), suggesting that these patterns develop early during postnatal development. Together, more than half of all randomly chosen adult AMCs displayed robust infra-slow oscillations in both cell-attached and whole-cell current-clamp recordings (Fig. 6*E*).

**Figure 7.**
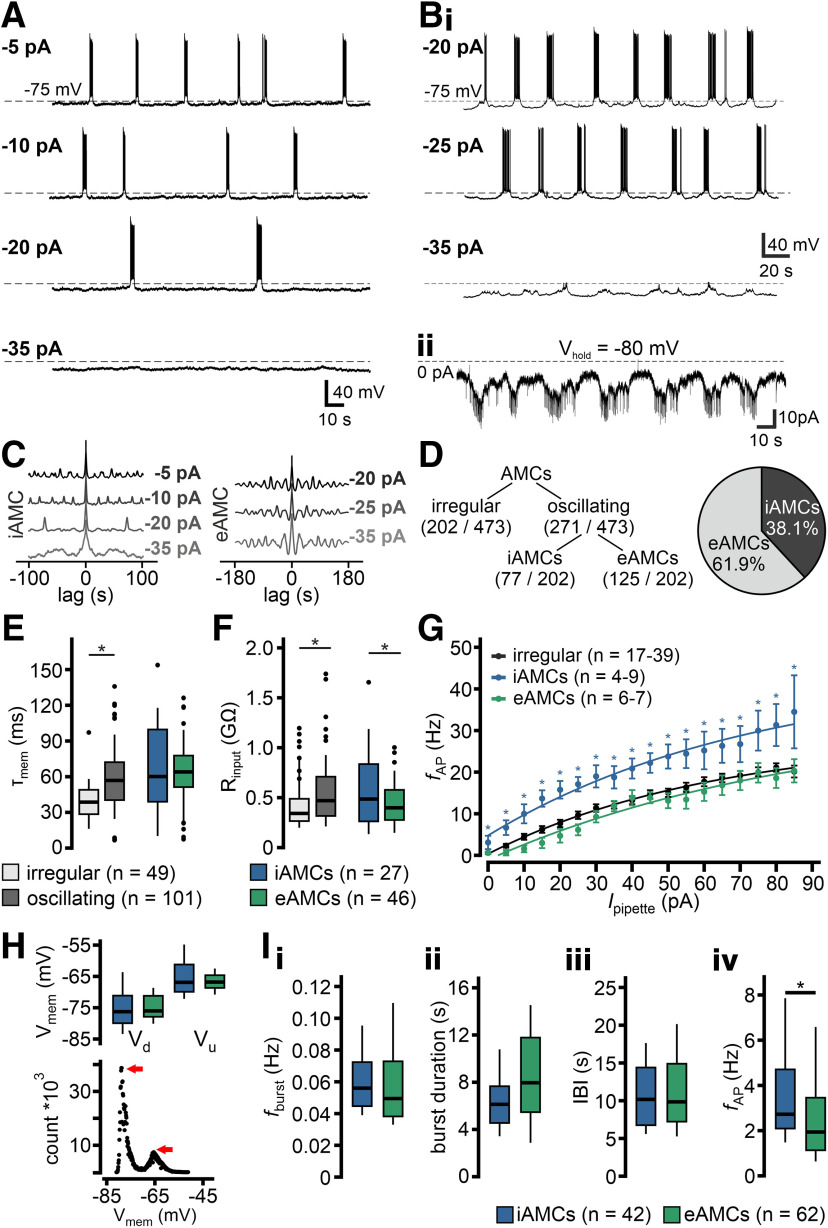
Continuous current injection reveals two populations of oscillating AMCs. ***A***, ***B***, Original whole-cell current-clamp recordings from two representative oscillating AMCs during continuous current injection of variable amplitude. Hyperpolarization increases IBIs in iAMCs (***A***). In this population, the pattern of periodically recurring “up” and “down” states switches to a stable resting state below a characteristic V_mem_ threshold (bottom). In another group of oscillating AMCs, the subthreshold oscillation is not affected by hyperpolarizing current injection (***B_i_***). Voltage-clamp recordings from such eAMCs (***B_ii_***) indicate that periodically occurring barrages of synaptic input likely mediate V_mem_ oscillations. ***C***, Auto-correlograms for the traces depicted in ***A*** and ***B_i_*** represent how oscillation frequency is affected as a function of current injection among iAMCs (left), whereas they remain qualitatively unaffected in eAMCs (right). ***D***, Oscillations are generated intrinsically in 38.1% of oscillating AMCs (77 of 202 cells). In the majority of AMCs (61.9%; 125 of 202 cells), oscillations are entrained. ***E***, ***F***, Box-and-whisker plots comparing τ_mem_ and R_input_, respectively. Boxes represent the first-to-third quartiles. Whiskers represent the 10th and 90th percentiles, respectively. Outliers (1.5 IQR) are plotted individually. The central band represents the population median (*P*_0.5_). Numbers of experiments are denoted in legends (bottom). Compared with irregularly active neurons, oscillating AMCs show an increased membrane time constant (***E***; 57.9 ± 2.6 vs 38.8 ± 2.4 ms, *p < 0.001; *P*_0.5_ = 56.9 vs 38.6 ms), and increased input resistance (***F***; 532.4 ± 30.9 vs 426.8 ± 36.2 MΩ, *p = 0.045; *P*_0.5_ = 470 vs 342.2 MΩ). While iAMCs and eAMCs show similar membrane time constants (***E***; 64.7 ± 7.0 vs 59.9 ± 3.8 ms; *P*_0.5_ = 57.0 vs 60.7 ms), iAMCs display increased input resistance (***F***; 608.8 ± 76.5 vs 445.4 ± 34.1 MΩ, *p = 0.03; *P*_0.5_ = 495.3 vs 406.1 MΩ). ***G***, *f*-*I* curves depicting average instantaneous discharge frequencies in irregular AMCs (black), iAMCs (blue), and eAMCs (green). Inset, Numbers of experiments. Maximum frequencies are 21.1 ± 1.0 Hz (irregular), 34.5 ± 8.8 Hz (iAMCs), and 20.3 ± 6.9 Hz (eAMCs), respectively. Individual data points are mean ± SEM. Curves are monoexponential fits. *Statistical significance between iAMCs and irregular AMCs (*p* < 0.02, unpaired *t* test). ***H***, Box-and-whisker plot (top) shows no substantial differences between iAMCs and eAMCs in either “down” state voltage (V_d_; −75.5 ± 1.2 vs −75.0 ± 0.6 mV; medians: −76.5 vs −76.0 mV) or “up” state membrane potential (V_u_; −65.7 ± 1.2 vs −66.9 ± 0.5 mV; medians: −66.9 vs −67.3 mV). Example all-points membrane potential histogram (bottom; 122 μV bin width) for an oscillating AMC that alternates between distinct “down” and “up” states (red arrows). ***I***, Box-and-whisker plots comparing oscillatory discharge parameters in iAMCs and eAMCs. Burst frequencies (***I_i_***; *f*_burst_) do not differ between iAMCs and eAMCs (*P*_0.5_ = 0.056 vs 0.050 Hz; 0.06 ± 0.02 vs 0.06 ± 0.06 Hz, mean ± SD). Similarly, no differences are apparent in either burst duration (***I_ii_***; *P*_0.5_ = 6.1 vs 8.0 s; 6.6 ± 2.8 vs 8.8 ± 5.1 s, mean ± SD) or IBI (***I_iii_***; *P*_0.5_ = 10.2 vs 9.6 s; 11.1 ± 5.1 vs 8.8 ± 5.1 s, mean ± SD). Within-burst spiking frequency (***I_iv_***; *f*_AP_), however, is significantly lower in eAMCs compared with intrinsically oscillating neurons (*P*_0.5_ = 1.9 vs 2.7 Hz; 2.7 ± 2.1 vs 3.7 ± 2.3 Hz (mean ± SD), *p = 0.027; unpaired *t* test).

Intrinsically rhythmogenic “pacemaker” neurons typically show a positive causal correlation between oscillation frequency and “baseline” V_mem_ (Crunelli and Hughes, 2010). Consequently, and as expected based on our previous results (Gorin et al., 2016), oscillation frequency changed as a function of hyperpolarizing current injection in several AMCs (Fig. 7*A*). Hyperpolarization increased, whereas depolarization reduced interburst intervals (IBIs) and these neurons exhibited a characteristic V_mem_ threshold below which the pattern of periodically recurring “up” and “down” states switched to a stable resting state (Fig. 7*A*). By contrast, other oscillating AMCs showed no correlation between V_mem_ and IBI (Fig. 7*Bi*). These neurons maintained infra-slow oscillatory V_mem_ fluctuations, even during subthreshold hyperpolarization with no change in subthreshold oscillation frequency (Fig. 7*C*). Moreover, voltage-clamp recordings from such AMCs indicated that V_mem_ oscillations are likely mediated by periodically occurring barrages of synaptic input (Fig. 7*Bii*). While single synaptic event parameters, such as postsynaptic current (PSC) amplitude, waveform, or charge transfer, did not differ between barrages and more “quiescent” IBIs, event frequency was significantly increased during bursts (data not shown). Thus, the quality/type of synaptic input appears to be the same during both bursts and interburst periods, and input frequency emerges as the determinant oscillatory drive. Our data, hence, suggest that fundamentally different mechanisms underlie oscillatory discharge in intrinsically rhythmogenic neurons (iAMCs) *versus* cells apparently entrained by the local network (eAMCs) (Fig. 7*C*,*D*). Upon volume-rendered 3D reconstruction and morphometric analysis of individual biocytin-filled neurons (*n* = 11, iAMCs; *n* = 27, eAMCs), the two physiologically distinct AMC populations displayed no obvious morphologic differences, neither with respect to surface area, nor to numbers of primary dendrites or glomerular tufts (data not shown). From here on, each oscillating neuron analyzed in patch-clamp recordings was initially categorized as either an iAMC or an eAMC by depolarizing/hyperpolarizing current injections and subsequent burst frequency analysis (Fig. 7*C*).

Among oscillating AMCs, which significantly differed from irregularly firing neurons in their passive membrane properties (Fig. 7*E*,*F*), iAMCs and eAMCs showed similar membrane time constants (Fig. 7*E*), but different input resistances (Fig. 7*F*). Notably, comparison of mean instantaneous spike frequencies as a function of stationary current input (*f*-*I* curves) revealed indistinguishable curves for both irregularly firing neurons and eAMCs, whereas iAMCs displayed increased excitability (Fig. 7*G*). This fact was previously overlooked when iAMCs and eAMCs (Gorin et al., 2016) were not distinguished. If iAMCs function as pacemaker neurons that entrain eAMCs, burst characteristics should be similar. This is indeed the case (Fig. 7*H*,*I*) as burst frequency (Fig. 7*Ii*), burst duration (Fig. 7*Iii*), and IBI (Fig. 7*Iiii*) did not substantially differ between the two AMC subpopulations. Confirming their generally increased excitability state (Fig. 7*G*), iAMCs exhibited significantly higher within-burst firing frequencies (Fig. 7*Iiv*). Together, our results show that the mouse AOB harbors a second population of oscillating neurons (eAMCs) that are less excitable than iAMCs, but receive periodic barrages of synaptic input that is independent of V_mem_.

**Figure 8.**
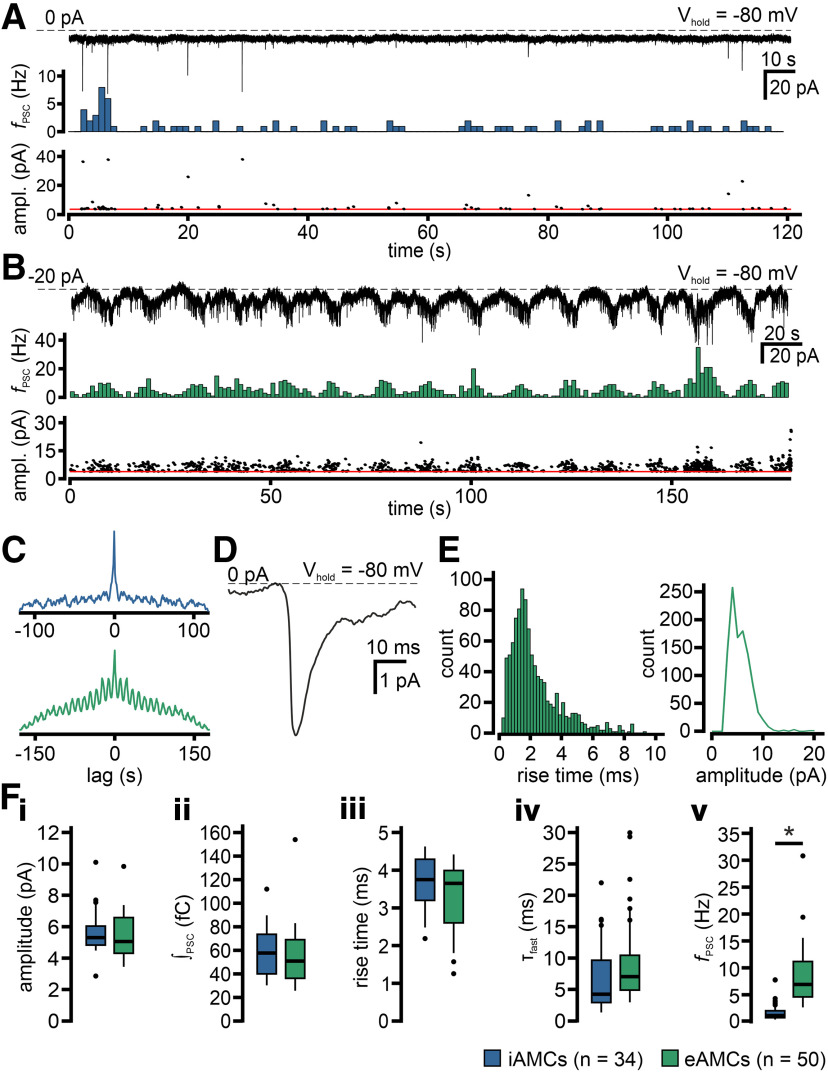
iAMCs and eAMCs receive different patterns of synaptic input. ***A***, ***B***, Representative whole-cell continuous voltage-clamp recordings (top; V_hold_ = −80 mV) of spontaneous PSCs in an iAMC (***A***) and eAMC (***B***), respectively. Note both the oscillation reflected in this example eAMC “baseline” current fluctuations (***B***) and the lack thereof in the representative iAMC (***A***). Traces display PSCs as downward deflections of varying amplitudes. PSC frequency histograms (middle; 1 s bin width; note different scaling between ***A*** and ***B***) illustrate synaptic input [mean frequency: 0.68 Hz (***A***) and 5.79 Hz (***B***)]. Corresponding amplitudes of individual PSCs (bottom) show relatively stable values over time [mean amplitude: 6.2 pA (***A***) and 6.1 pA (***B***)]. Red horizontal lines indicate detection thresholds at 3.5 pA (***A***) and 3.7 pA (***B***), respectively. ***C***, Auto-correlation histograms constructed from the original recordings from the iAMC (blue) and eAMC (green) shown in ***A*** and ***B*** reveal rhythmicity (or lack thereof). ***D***, Average waveform of all detected events in ***B***. ***E***, Rise time (left; 0.2 ms bin width) and amplitude (right; 1 pA bin width) histograms of events detected in ***B***. ***F***, Quantification of synaptic input to iAMCs (blue) and eAMCs (green). Box-and-whisker plots comparing spontaneous PSC amplitudes (***F_i_***), charge transfer (***F_ii_***; ∫_PSC_), rise times (***F_iii_***), decay constants (***F_iv_***; τ_fast_), and frequencies (***F_v_***; *f*_PSC_), respectively. Boxes represent the first-to-third quartiles. Whiskers represent the 10th and 90th percentiles, respectively. The central band represents the population median (*P*_0.5_). No differences between iAMCs and eAMCs are found in PSC amplitude (5.4 ± 0.2 vs 5.6 ± 0.2 pA; *P*_0.5_ = 5.1 vs 5.3 pA), charge transfer (−54.1 ± 3.6 vs −58.7 ± 4.3 fC; *P*_0.5_ = −50.9 vs −57.7 fC), rise time (2.9 ± 0.1 vs 3.3 ± 0.2 ms; *P*_0.5_ = 2.9 vs 3.4 ms), or τ_fast_ (8.6 ± 0.9 vs 6.6 ± 1.1 ms; *P*_0.5_ = 7.0 vs 4.2 ms). The frequency of synaptic input, however, is significantly increased in eAMCs compared with iAMCs (10.3 ± 1.4 vs 1.9 ± 0.4 Hz; *P*_0.5_ = 6.9 vs 1.1 Hz; *p* < 0.001; unpaired *t* test).

Next, we asked whether and, if so, how synaptic input differs between entrained and intrinsically rhythmogenic AOB neurons. Voltage-clamp recordings revealed that, compared with entrained neurons, iAMCs received considerably less input (Fig. 8*A*,*B*). Moreover, iAMC synaptic currents mostly lacked obvious periodicity, whereas input rhythmicity was pronounced in eAMCs (Fig. 8*C*). PSCs showed rise times of several milliseconds (Fig. 8*D*,*E*) and, on average, PSC shape was indistinguishable between eAMCs and iAMCs (Fig. 8*Fi-iv*). Input frequency, by contrast, was markedly increased in eAMCs (Fig. 8*Fv*). Overall, our data indicate that iAMCs and eAMCs receive qualitatively similar input. While this input is irregular and rather sparse in intrinsically rhythmogenic neurons, eAMCs receive and are likely driven by periodically recurring barrages of PSCs.

### Synaptic entrainment comes in two flavors: glutamate-dependent and -independent excitation

**Figure 9.**
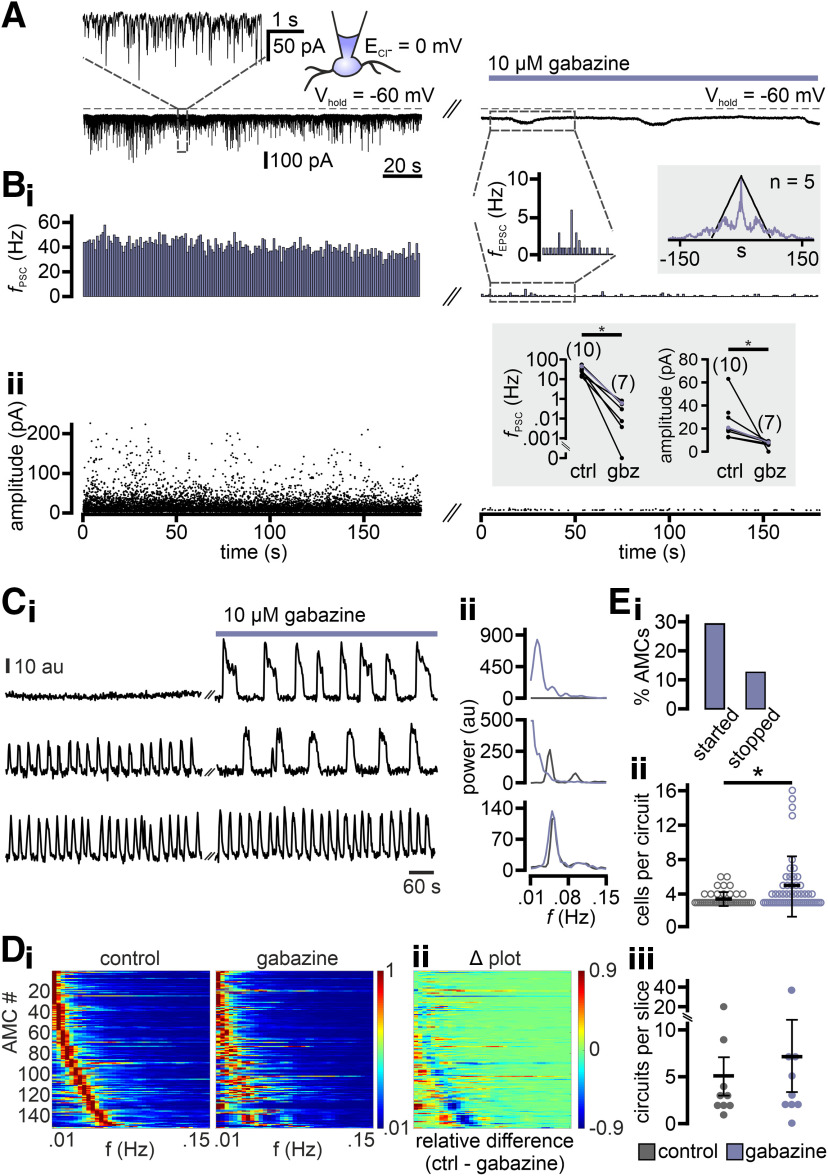
Nonperiodic inhibitory synaptic input alters AMC oscillatory activity. ***A***, Continuous whole-cell voltage-clamp recording (V_hold_ = −60 mV) of representative spontaneous inhibitory PSCs [downward deflections of varying amplitudes; chloride equilibrium potential (E_Cl_, inset) shifted to 0 mV]. AMCs receive extensive inhibitory synaptic input under control conditions (left). Dashed rectangle represents segment displayed at enlarged temporal coordinates above. Inhibition of fast GABAergic transmission (right; 10 μm gabazine) strongly reduces the number of detected PSCs per 3 min recording [*n* = 7289 (control) vs 98 (gabazine)]. Periodic “baseline” deflections (masked by inhibitory PSCs under control conditions) suggest that this recording was obtained from an oscillating AMC. ***B***, PSC frequency histogram (***B_i_***; 1 s bin width) and amplitude plot (***B_ii_***; detection threshold: 3 pA) during control conditions (left) and gabazine treatment (right; 10 μm). In absence of pharmacological agents, AMCs receive robust, tonic synaptic input (average *f*_PSC_ = 40.5 Hz). The lack of PSC periodicity is evident from the auto-correlation histogram of detected events (top right inset; black; 1s bin width). By contrast, those PSCs that remain in presence of gabazine (average *f*_PSC_ = 0.5 Hz) do occur periodically (top right inset; violet). When the frequency histogram is shown on an expanded *y* axis (top left inset), the transient increase in EPSC frequency during baseline deflections becomes apparent. Moreover, PSC amplitudes are strongly diminished upon inhibition of GABAergic transmission (***B_ii_***). Bottom inset, Pairwise quantification of both PSC frequency (left) and amplitude (right) to a mixed group of AMCs (numbers of experiments as indicated). Data points corresponding to the recording shown in ***A*** are highlighted (violet). Both PSC parameters are significantly reduced upon gabazine treatment [*f*_PSC_ = 9.4 ± 3.8 Hz (control) vs 0.3 ± 0.1 Hz (gabazine), *p* < 0.0001; amplitude = 24.1 ± 4.9 pA (control) vs 6.7 ± 1.2 pA (gabazine), *p* = 0.01]. *Statistical significance (unpaired *t* tests). ***C***, Representative recordings of average fluorescence intensities (au, arbitrary units) over time illustrate the three main types of AMC activity in response to gabazine treatment. Ten minute recordings are shown before and after drug incubation, respectively (***C_i_***). Effects (or the lack thereof) also become apparent in the corresponding power spectra (***C_ii_***), where colors represent control (black) or treatment conditions (violet). Block of GABAergic inhibition either triggers periodic activity in previously “silent” AMCs (top row), slows oscillations (middle row), or has essentially no effect (bottom row). ***D***, Heat maps represent normalized power spectra for a total of 153 oscillating AMCs before and during gabazine treatment (***D_i_***). Individual spectra are aligned according to the lowest peak frequency under control conditions. ***D_ii_***, Changes become visible in a Δ heat map. Shifts in spectral power as relative differences between both conditions. ***E***, Quantitative analysis of gabazine-mediated changes in AMC phenotype and microcircuit formation. ***E_i_***, Bar graph represents the fractions of neurons that either started to display rhythmic activity (29.2%) or ceased to show such bursts (12.6%) after block of GABAergic inhibition. Upon gabazine treatment, the number of AMCs that constitute a microcircuit (***E_ii_***) is significantly increased (*p* < 0.05; Wilcoxon rank sum test), whereas the number of microcircuits per AOB slice (***E_iii_***) remains essentially unchanged (*p* = 0.86; Wilcoxon signed rank test).

To investigate the nature of the synaptic input that drives eAMC oscillations, we first asked what role, if any, is played by GABAergic inhibition. Previous recordings (Fig. 8) were performed at a holding potential (V_hold_ = −80 mV) relatively close to the calculated equilibrium potential for Cl^–^ (E_Cl_ = −59 mV), suggesting that recorded PSCs are mostly excitatory. After shifting E_Cl_ to 0 mV, we recorded pronounced high-frequency inward currents that were sensitive to the GABA_A_ receptor antagonist gabazine (Fig. 9*A*,*B*). These GABAergic synaptic currents showed no sign of periodicity. Rather, excitatory current rhythmicity was “unmasked” in some cells by gabazine treatment, as evident from corresponding auto-correlograms (Fig. 9*B*, top inset). Thus, patch-clamp recordings indicate that AMCs receive relatively constant levels of GABAergic inhibition *in vitro*.

Next, we asked whether indeed a constant GABAergic inhibitory tone affects oscillatory AMC discharge. Confocal population Ca^2+^ imaging of GCaMP6f-expressing AMCs reveals that gabazine treatment affects oscillatory signaling in the vast majority of neurons (Fig. 9*C*,*D*). In most oscillating AMCs, block of fast GABAergic synaptic transmission resulted in reduced burst frequencies as evident from spectral analysis (Fig. 9*Dii*). Notably, a substantial AMC fraction only began to oscillate after gabazine treatment (Fig. 9*C*,*Ei*). If excitatory drive underlies microcircuit assembly, one would expect more AMCs to be recruited into synchronized ensembles in the absence of inhibition and the corresponding disruption of excitation-inhibition balance. This is indeed the case (Fig. 9*Eii*). The number of circuits per slice, however, remained unaltered (Fig. 9*Eiii*), indicating that removal of GABAergic inhibitory tone does not unmask potentially “silent” microcircuits.

**Figure 10.**
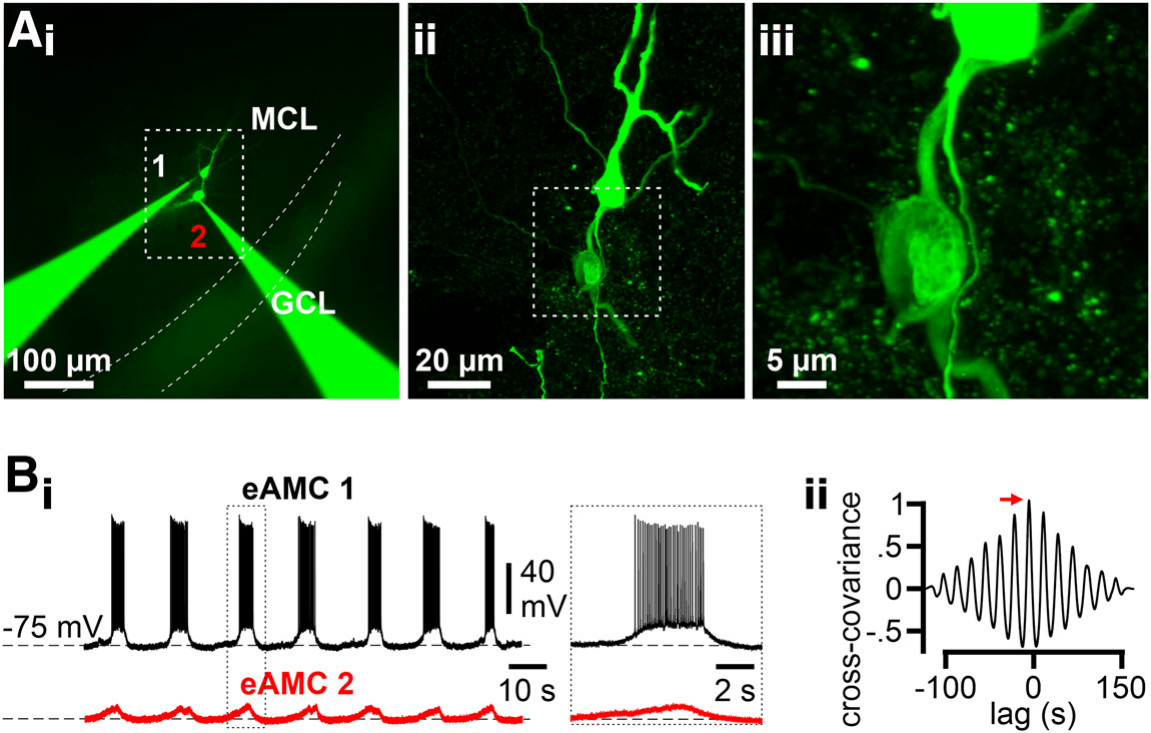
Paired whole-cell patch-clamp recordings from randomly chosen AMCs reveal that direct coupling, if existent, is sparse. ***A***, Images represent two AMCs from which simultaneous intracellular recordings were performed. ***A_i_***, Wide-field epifluorescence photomicrograph. Both AMCs were diffusion-loaded via the two patch pipettes with Alexa-488 hydrazide and biocytin. GCL, Granule cell layer; MCL, mitral cell layer. ***A_ii_***, Maximum projection of a confocal *z* stack of the dashed box in ***A_i_*** after *post hoc* streptavidin labeling. ***A_iii_***, Enlarged view of the area delimited by the dashed box in ***A_ii_*** depicts the two AMC somata and proximal dendrites. ***B_i_***, Example paired current-clamp recordings (***B_i_***) from the two AMCs shown in ***A***. Period outlined by dashed rectangle is shown on an expanded time scale. Burst firing in eAMC 1 coincides with subthreshold depolarization in eAMC 2. ***B_ii_***, Normalized cross-covariance plot (***B_ii_***) shows correlated, but phase-shifted, signals (365 ms shift; red arrow).

One potential mechanism of functional AMC coupling is electrical connectivity via gap junctions (Zylbertal et al., 2017). Given the poor specificity of all pharmacological agents available to study gap junctions (Beaumont and Maccaferri, 2011; Connors, 2012), we opted for direct electrical coupling analysis via paired patch-clamp recordings. Using standard protocols (Debanne et al., 2008), we recorded from a total of 18 AMC pairs of various subtype combinations (Fig. 10), eight of which were tested reciprocally. We found no clear evidence for direct electrical connectivity (data not shown). While this does not rule out a role of gap junctions in AMC microcircuit formation, our results show that electrical coupling, if existent, must be relatively sparse. This is not unexpected given our AMC population imaging results (Figs. 3–5). While we usually recorded from >20 AMCs in a given slice (23.6 ± 16.5; mean ± SD; *n* = 43), the average microcircuit (per confocal plane) consisted of 3.3 ± 0.7 neurons (73 circuits). Therefore, the probability of picking a pair of synchronized ensemble members by chance is ∼1.4%.

**Figure 11.**
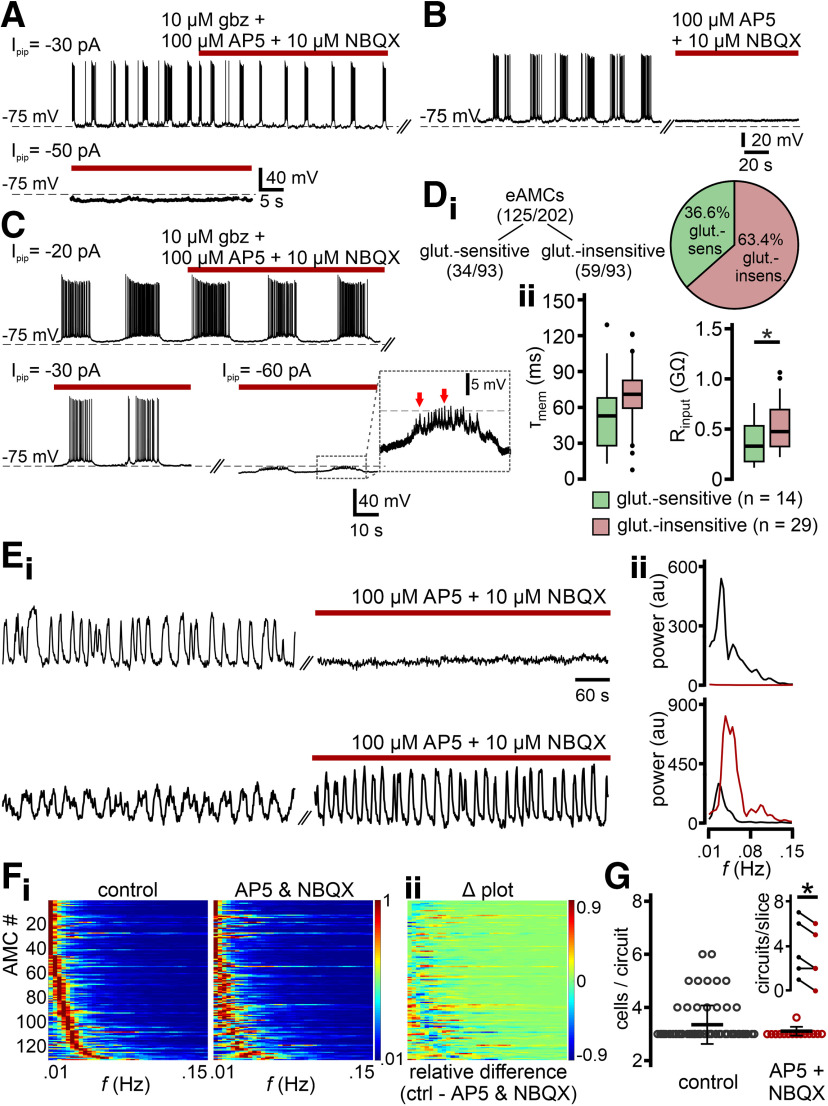
Pharmacological profiles distinguish two eAMC populations. ***A-C***, Original whole-cell current-clamp recordings from three representative AMC types. ***A***, In iAMCs, oscillations persist during synaptic isolation (top; gabazine + NBQX + AP5). Moreover, a characteristic switch from a bistable membrane potential to a stable resting state is observed upon hyperpolarization (bottom). ***B***, In a second population of oscillatory AMCs, inhibition of fast excitatory transmission (AP5 + NBQX) abolishes both rhythmic discharge and subthreshold V_mem_ oscillations. ***C***, Combining continuous hyperpolarizing current injections of varying amplitudes (−30 to −60 pA) with pharmacological inhibition of fast synaptic transmission (gabazine + NBQX + AP5) reveals a third oscillatory AMC population. In this group, oscillations are insensitive to synaptic isolation (top). Hyperpolarization, however, does not affect oscillation frequency (bottom), even at subthreshold V_mem_. Note barrages of depolarizing postsynaptic potentials during “up” states (red arrows) in expanded view. ***D***, eAMCs segregate into two distinct subpopulations (pie chart). ***D_i_***, In one group, oscillations are sensitive to block of fast glutamatergic transmission (36.6%; 34 of 93 cells). In a second eAMC population, however, network-dependent oscillations remain unaffected by inhibition of glutamatergic transmission (63.4%; 59 of 93 cells). ***D_ii_***, Box-and-whisker plots comparing τ_mem_ (left) and R_input_ (right) between glutamate-sensitive (green; *n* = 14) and -insensitive (magenta; *n* = 29) eAMCs. Boxes span the first-to-third quartiles. Whiskers represent the 10th and 90th percentiles, respectively. Outliers (1.5 IQR) are plotted individually. The central band represents the population median (*P*_0.5_). While membrane time constants are statistically indifferent (48.9 ± 7.6 vs 65.3 ± 4.4 ms; *P*_0.5_: 49.0 vs 65.8 ms; *p* = 0.055, unpaired *t* test), input resistance is significantly increased in glutamate-insensitive neurons (350.2 ± 56.0 vs 502.9 ± 44.0 MΩ; *P*_0.5_: 317.1 vs 456.7 MΩ; *p < 0.05, unpaired *t* test). ***E***, Representative confocal recordings of average GCaMP6f fluorescence intensities (au, arbitrary units) over time illustrate the two main types of AMC activity in response to AP5/NBQX treatment. Ca^2+^ traces recorded during population imaging experiments (10 min) illustrate activity before and after drug incubation, respectively (***E_i_***). Effects also become apparent in the corresponding power spectra (***E_ii_***). Block of fast glutamatergic transmission either silences AMCs (top; observed in 18.8% of neurons) or substantially changes oscillation patterns (bottom). ***F***, Heat maps illustrate normalized power spectra of 134 AMCs that oscillate before and during inhibition of fast glutamatergic synaptic transmission (***F_i_***). Individual spectra are aligned according to the lowest frequency band with peak power under control conditions. Changes in periodicity become visible in a Δ heat map (***F_ii_***), illustrating shifts in spectral power as relative differences between both conditions. ***G***, Quantitative analysis of AP5/NBQX-mediated changes in AMC microcircuit formation. Dot plots (including mean ± SD) show that the number of AMCs that constitute a microcircuit appears reduced upon inhibition of ionotropic glutamate receptors [3.4 ± 0.7 (control; *n* = 70); 3.1 ± 0.3 (AP5/NBQX; *n* = 15)]. Moreover, the number of microcircuits per AOB slice is significantly reduced after drug treatment (inset; *p < 0.01, paired *t* test).

Next, we asked whether fast glutamatergic input drives eAMC oscillations. As previously described (Gorin et al., 2016), iAMCs were unaffected by block of both AMPA/kainate and NMDA receptors in whole-cell current-clamp recordings (Fig. 11*A*). By contrast, burst firing ceased in a substantial fraction of eAMCs after block of fast glutamatergic transmission by AP5 and NBQX (Fig. 11*B*). Surprisingly, many eAMCs remained unperturbed by pharmacological treatment (Fig. 11*C*). These results show that eAMCs comprise at least two subpopulations of AOB projection neurons: one driven by periodic barrages of glutamatergic input and another that is entrained independently of AMPA/kainate and NMDA receptor activation (Fig. 11*D_i_*). Notably, glutamate-sensitive and -insensitive eAMCs differ regarding their membrane input resistance and, thus, their excitability, with glutamate-insensitive eAMCs exhibiting higher input resistance (Fig. 11*Dii*). The two general AMC subtypes also became apparent in population Ca^2+^ imaging recordings (Fig. 11*E*). Among those neurons that maintained oscillatory activity during AP5/NBQX treatment (i.e., either iAMCs or glutamate-insensitive eAMCs), we often observed a qualitative change in the power spectrum (Fig. 11*F*). Importantly, block of glutamatergic transmission reduced both the number of cells that constitute a microcircuit and the number of circuits found per slice (Fig. 11*G*). These results show that fast glutamatergic drive is an important, but not the sole mechanism involved in AMC microcircuit assembly.

**Figure 12.**
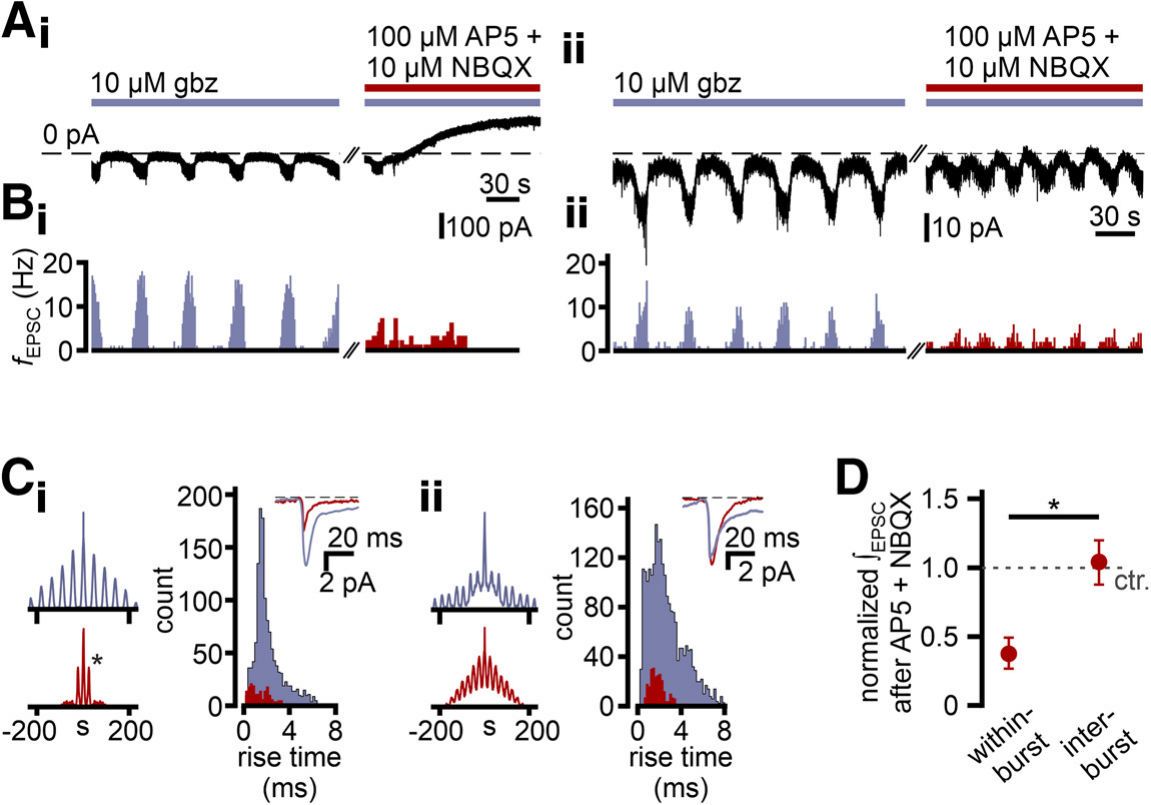
Excitatory input to eAMCs is rhythmic and drives oscillations. ***A***, Representative continuous voltage-clamp recording (V_hold_ = −75 mV) of spontaneous EPSCs (downward deflections of varying amplitudes) in a glutamate-sensitive (***A_i_***) and a glutamate-insensitive (***A_ii_***) eAMC, recorded during gabazine treatment. Corresponding to burst firing epochs observed in V_mem_ recordings, EPSCs are situated on oscillating baseline inward current deflections. Inhibition of fast glutamatergic transmission (AP5 + NBQX) abolishes oscillations in glutamate-sensitive eAMCs (***A_i_***). Note the baseline shift to positive stationary currents, indicating V_mem_ hyperpolarization. ***B***, EPSC frequency (*f*_EPSC_) histograms (1 s bin width) demonstrate that, during gabazine incubation (violet), excitatory synaptic input predominantly occurs in rhythmic barrages in both glutamate-sensitive (***B_i_***) and glutamate-insensitive (***B_ii_***) eAMCs. Isolation from fast glutamatergic transmission (red) abolishes EPSC rhythmicity in glutamate-sensitive eAMCs (***B_i_***). By contrast, while AP5/NBQX also reduces *f*_EPSC_ in glutamate-insensitive eAMCs (***B_ii_***), rhythmicity remains. ***C***, Auto-correlograms (1 s bin width) constructed from recordings shown in ***A***. Color code represents gabazine (violet) and gabazine/AP5/NBQX (red) treatment. The singular side peak (***C_i_***, *, red) results from the sole EPSC burst that coincides with the start of gabazine/AP5/NBQX treatment. By contrast, periodicity of excitatory input to glutamate-insensitive eAMCs is unaffected (***C_ii_***). Insets, Averaged EPSCs (aligned at half-rise) for each pharmacological condition. EPSC rise time histograms (0.2 ms bin width) indicate that synaptic currents insensitive to gabazine/AP5/NBQX develop fast, with rise times generally <4 ms. ***D***, Relative to control conditions (10 μm gabazine; *n* = 10), charge transfer is substantially reduced upon inhibition of ionotropic glutamate receptors (100 μm AP5 + 10 μm NBQX; *n* = 4) during bursts (0.38 ± 0.11), but largely unaffected during more quiescent IBIs (1.07 ± 0.14).

Given the two categorically different effects AP5/NBQX treatment exerted on eAMC oscillatory discharge, we asked whether this functional dichotomy is reflected in the PSC. Indeed, block of fast glutamatergic transmission essentially abolished rhythmic synaptic input in some neurons (Fig. 12*Ai–Ci*), whereas periodic barrages of PSCs appeared largely unperturbed in other cells (Fig. 12*Aii–Cii*). In the latter group, however, AP5/NBQX did, indeed, also reduce PSC frequency strongly, but periodicity remained unaltered (Fig. 12*Cii*). Surprisingly, isolation from fast excitatory synaptic transmission (AP5 and NBQX) in glutamate-sensitive eAMCs selectively reduced charge transfer during periods of high postsynaptic activity (Fig. 12*D*), whereas the comparably low input level between bursts remained essentially unaltered.

Together, these data strengthen our conclusion that relatively sparse feedforward excitation (both glutamate-dependent and -independent) is a major mechanism underlying the assembly of AMC microcircuits.

## Discussion

The accessory olfactory system is central to social information processing. Surprisingly, however, many physiological principles underlying AOB sensory processing remain poorly understood (Dulac and Wagner, 2006). We and others (Gorin et al., 2016; Vargas-Barroso et al., 2016; Zylbertal et al., 2017) recently observed that slow to infra-slow oscillations represent the default activity pattern of some AMCs *in vitro*. Here, we report that such infra-slow stereotypical rhythmic activity also characterizes the idle state of at least some AMC ensembles in awake unrestrained mice. Notably, and most likely resulting from dendritic action potential backpropagation (Ma and Lowe, 2004), oscillations emerge on the glomerular scale, strongly suggesting a role in information processing. Individual glomeruli display distinct rhythmicity, a fact mirrored by synchronous *in vitro* activity among AMC ensembles. These parallel microcircuits likely contain both intrinsically rhythmogenic neurons and AMCs that are entrained by periodic barrages of excitatory synaptic input. Some, but not all, synaptic entrainment is driven by glutamate and likely involves feedforward excitation. Together, our findings establish infra-slow synchronous oscillatory activity within distinct AMC microcircuits as a physiologically relevant phenomenon that adds new dimension(s) to chemosensory coding along the accessory olfactory pathway.

The default activity pattern of any neuronal network emerges from the combination of the constituent neurons' intrinsic electrical characteristics and their synaptic wiring (Stagkourakis et al., 2018). Among AMCs, oscillation frequencies are highly heterogeneous (Gorin et al., 2016; Zylbertal et al., 2017). Coexistence of iAMCs and eAMCs, which both span a wide and overlapping frequency spectrum, supports the notion of parallel microcircuit formation by pacemaker-like activity of phenotypically different iAMCs that bind groups of eAMCs into synchronous ensembles. Prominent slow oscillations are generated by similar circuit configurations in neocortex and thalamus during inattentive wakefulness and non-REM sleep (Crunelli et al., 2018). In addition, astrocytes, which constitute an abundant glial subtype in the rodent olfactory bulb (Bailey and Shipley, 1993), could exert profound effects on the generation of AOB oscillations. Astrocytic modulation of excitability through K^+^ spatial buffering (Verkhratsky and Nedergaard, 2018; Buskila et al., 2019) has been shown to control network formation and synchrony (Ding et al., 2016) as well as state transitions (Diaz Verdugo et al., 2019). Another mechanism that has been shown to shape infra-slow oscillations in thalamic networks is based on adenosine A1 receptor activation by ATP-derived adenosine (Lorincz et al., 2009). Treatment of acute AOB slices with the A1 receptor antagonist DPCPX (2 μm), however, had essentially no effect on AMC rhythmicity (data not shown).

While the AOB harbors reciprocal dendrodendritic synapses between mitral and granule cell dendrites (Hayashi et al., 1993; Jia et al., 1999), GABAergic synaptic inhibition does not cause oscillatory discharge, confirming previous observations (Zylbertal et al., 2017). Indeed, during the hyperpolarized “down” state, we observed extreme paucity of excitatory synaptic activity. In sharp contrast to a recent report by Zylbertal et al. (2017), however, we do not find that GABA_A_ receptor block results in complete synchrony of the entire AOB neural population. While blocking fast inhibitory transmission induces oscillatory activity in some AMCs, formation of parallel microcircuits remains unchanged. These discrepancies could simply stem from a different conception of “synchrony” (Zylbertal and coworkes allowed ±15 s lag in maximum correlation between “synchronous” cell pairs) and/or fundamentally different definitions of an assembly (Zylbertal and coworkes did not require each assembly member to be correlated with all remaining cells). Alternatively, differences could have methodological reasons (i.e., wide-field *vs* confocal imaging).

Proximity is no requirement for participation in a microcircuit (Figs. 4*F*, 5*D*). Indeed, we sometimes found correlated activity in neurons located along almost the entire rostrocaudal AOB axis. This is noteworthy as AMC dendrites respect the two major AOB subdivisions (i.e., a given AMC samples from either the rostral or caudal glomerular subdivisions) (Belluscio et al., 1999; Del Punta et al., 2002). However, AMC somata are not necessarily located in the same divisions as their glomerular dendrites (Yonekura and Yokoi, 2008). Therefore, it remains to be investigated whether all members of a given microcircuit extend their glomerular dendrites within the same AOB subdivision, potentially targeting overlapping or even identical glomerular subsets (see below). While members of a given ensemble are not spatially clustered along the AOB rostrocaudal axis, clustering perpendicular to the optical section plane cannot be excluded.

Based on modeling and pharmacology, others have recently proposed a prominent role of gap junction coupling in correlated AMC activity (Zylbertal et al., 2017). While we cannot exclude a possible function of electrical synapses in microcircuit formation, we are aware of the profound methodological limitations in studying gap junctions. Pharmacological agents are compromised by poor specificity (Beaumont and Maccaferri, 2011; Connors, 2012), genetic animal models often exhibit incomplete loss of function (Fenno et al., 2014), and similar oscillatory phenotypes have been shown both with and without gap junctions (De Zeeuw et al., 2003; Crunelli et al., 2018; Stagkourakis et al., 2018). Our findings instead point to a key role of fast excitatory synaptic connectivity in AOB microcircuit formation, whereas neither feedforward nor feedback inhibition appears to play a major role (Buzsáki, 2006). Indeed, depolarizing envelopes were associated with barrages of excitatory synaptic inputs, whereas silent interburst periods showed a marked withdrawal of such inputs. While not exclusively responsible for microcircuit formation, both AMPA/kainate and NMDA receptors are major factors in synchronous ensemble activity. Whether AMC coupling is direct (i.e., monosynaptic) or indirect (polysynaptic) is currently unclear. Paired patch-clamp recordings indicate that direct coupling between AMCs, via gap junctions and/or chemical synapses, is rare.

While *in vivo* microcircuit activity is most likely subject to centrifugal top-down modulation (Mohedano-Moriano et al., 2012; In 't Zandt et al., 2019), slice experiments demonstrate that the AOB network itself is sufficient for ensemble formation and oscillatory discharge. However, experimental *in vitro* conditions might favor oscillatory activity, whereas both peripheral sensory input and top-down modulation could add substantial “noise” (Mohrhardt et al., 2018), which could also explain any apparent variation in periodicity “strength” between *in vitro* and *in vivo* recordings. Using extracellular single-unit recordings in anesthetized animals, we recently reported rhythmic bursting in 12% of all units *in vivo* (Gorin et al., 2016). Here, we show that AMC oscillatory activity translates to the level of individual glomeruli in awake mice. This finding suggests that (1) those AMCs that constitute a microcircuit may also target a common set of glomeruli, and (2) synchronous activity within an ensemble/glomerulus dominates each microcircuit's idle state *in vivo*. Since vomeronasal sensory neurons exhibit spontaneous burst firing at variable frequencies (Arnson and Holy, 2011), it is conceivable that peripheral input also plays an entraining role in ensemble formation.

Baseline AMC rhythmicity will have considerable physiological impact on sensory processing along the accessory olfactory pathway (Mohrhardt et al., 2018). As shown previously (Ma and Lowe, 2004; Urban and Castro, 2005) and as deduced from oscillatory Ca^2+^ signals in individual glomeruli *in vivo*, AMC dendrites actively backpropagate signals from the soma to the glomerular tufts. Thus, rhythmically alternating postsynaptic excitability might provide “windows of opportunity” (Buzsáki, 2006; Schaefer et al., 2006) for effective signal transmission and integration not only at the output stage, but already at the input level (Fries, 2015). Rhythmic cycles between high and low postsynaptic excitability states add a novel temporal dimension to the system's sensory coding space (Schroeder and Lakatos, 2009). Since AOB responses are generally slow (Shpak et al., 2012; Yoles-Frenkel et al., 2018), in terms of both onset time and duration, they fit the temporal scale on which periodic AMC discharge operates to modulate synaptic input gain. Given the prolonged activity during “up” states and the infra-slow cycle between the “up” and “down” state, correlated activity within an AMC ensemble does not necessarily demand millisecond precision to entail physiologically relevant synchrony. Therefore, even relatively small, yet significant correlations among coupled AMCs willset individual ensembles apart fromthe general AMC population. Notably, downstream processing modules include several nuclei that mediate slow pulsatile neuroendocrine release by synchronized slow rhythmic bursting of, for example, GnRH (Chu et al., 2012) or vasopressin (Brown, 2004) neurons on comparable time scales.

In contrast to intrinsic theta oscillations in main olfactory bulb external tufted cells (Hayar et al., 2004, 2005), which entrain to the sniffing cycle (Cury and Uchida, 2010), the heterogeneity in oscillation frequencies among AMC microcircuits argues against a similar entrainment by, for example, the vomeronasal pump (Meredith and O'Connell, 1979). However, knowledge about the operation of the peristaltic pump in the mouse vomeronasal organ is fragmentary at best. While functional links between peripheral vasoconstriction cycles and AMC periodic *in vivo* activity can thus not be ruled out, the robust oscillations that occur in AOB slices demonstrate that vomeronasal pumping is not required.

Microcircuit formation and synchronous oscillatory discharge increase the AOB's coding capacity. Coherent oscillatory ensemble activity could both facilitate input selection (Buzsáki and Draguhn, 2004) and ensure dynamic gating, reliability, and selectivity of communication (Izhikevich et al., 2003; Buzsáki et al., 2013) between the AOB and downstream networks. Inputs that arrive at moments of high input gain benefit from enhanced effective connectivity (Fries, 2015). In addition, bursts add reliability to signal transmission (Zeldenrust et al., 2018), as they are less sensitive to noise (Crunelli et al., 2018). Slow rhythms, in particular, can reset and temporally bias local computation (Buzsáki et al., 2013), which can in turn induce comodulation of the power of faster oscillations (Buzsáki and Wang, 2012). Our data confirm that, both *in vitro* and *in vivo*, diverse rhythms can coexist in the activity of a single neuron (Sirota et al., 2008). Several exciting questions remain to be addressed. Are infra-slow AMC microcircuit oscillations state-dependent? How are oscillations affected by sensory input? What are the downstream target neurons of synchronized AMC ensembles? Future efforts to answer these questions will deepen our conceptual understanding of sensory information processing in the accessory olfactory system.

In conclusion, we identify infra-slow periodic patterns of concerted neural activity within distinct sets of AOB glomeruli *in vivo*. These patterns most likely result from correlated discharge oscillations among groups of AMCs that assemble into parallel microcircuits. Ensemble formation is based on intrinsically rhythmogenic AMCs and neurons entrained by fast synaptic input.
